# A multi-omic landscape of steatosis-to-NASH progression

**DOI:** 10.1093/lifemeta/loac034

**Published:** 2022-12-01

**Authors:** Liping Xiang, Xiaoyan Li, Yunchen Luo, Bing Zhou, Yuejun Liu, Yao Li, Duojiao Wu, Lijing Jia, Pei-Wu Zhu, Ming-Hua Zheng, Hua Wang, Yan Lu

**Affiliations:** Department of Endocrinology and Metabolism, Shanghai Clinical Center for Diabetes, Shanghai Diabetes Institute, Shanghai Key Laboratory of Diabetes Mellitus, Shanghai Sixth People’s Hospital Affiliated to Shanghai Jiao Tong University School of Medicine, Shanghai, China; Key Laboratory of Metabolism and Molecular Medicine of the Ministry of Education, Department of Endocrinology and Metabolism, Zhongshan Hospital, Fudan University, Shanghai, China; Department of Oncology, the First Affiliated Hospital of Anhui Medical University, Hefei, China; Department of Endocrinology and Metabolism, Shanghai General Hospital, Shanghai Jiao Tong University School of Medicine, Shanghai, China; Department of Endocrinology and Metabolism, Shanghai Clinical Center for Diabetes, Shanghai Diabetes Institute, Shanghai Key Laboratory of Diabetes Mellitus, Shanghai Sixth People’s Hospital Affiliated to Shanghai Jiao Tong University School of Medicine, Shanghai, China; Key Laboratory of Metabolism and Molecular Medicine of the Ministry of Education, Department of Endocrinology and Metabolism, Zhongshan Hospital, Fudan University, Shanghai, China; Department of Laboratory Animal Science, Shanghai Jiao Tong University School of Medicine, Shanghai, China; Zhongshan Hospital Institute of Clinical Science, Fudan University, Shanghai Institute of Clinical Bioinformatics, Shanghai, China; Department of Endocrinology, The Second Clinical Medical College, Jinan University (Shenzhen People’s Hospital), Shenzhen, China; Department of Laboratory Medicine, First Affiliated Hospital of Wenzhou Medical University, Wenzhou, Zhejiang, China; MAFLD Research Center, Department of Hepatology, The First Affiliated Hospital of Wenzhou Medical University, Wenzhou, Zhejiang, China; Department of Oncology, the First Affiliated Hospital of Anhui Medical University, Hefei, China; Institute of Metabolism and Regenerative Medicine, Shanghai Sixth People’s Hospital Affiliated to Shanghai Jiao Tong University School of Medicine, Shanghai, China

**Keywords:** multi-omics, nonalcoholic steatohepatitis, simple steatosis, GDF3, ferroptosis

## Abstract

Nonalcoholic steatohepatitis (NASH) has emerged as a major cause of liver failure and hepatocellular carcinoma. Investigation into the molecular mechanisms that underlie steatosis-to-NASH progression is key to understanding the development of NASH pathophysiology. Here, we present comprehensive multi-omic profiles of preclinical animal models to identify genes, non-coding RNAs, proteins, and plasma metabolites involved in this progression. In particular, by transcriptomics analysis, we identified Growth Differentiation Factor 3 (GDF3) as a candidate noninvasive biomarker in NASH. Plasma GDF3 levels are associated with hepatic pathological features in patients with NASH, and differences in these levels provide a high diagnostic accuracy of NASH diagnosis (AUROC = 0.90; 95% confidence interval: 0.85−0.95) with a good sensitivity (90.7%) and specificity (86.4%). In addition, by developing integrated proteomic-metabolomic datasets and performing a subsequent pharmacological intervention in a mouse model of NASH, we show that ferroptosis may be a potential target to treat NASH. Moreover, by using competing endogenous RNAs network analysis, we found that several miRNAs, including miR-582-5p and miR-292a-3p, and lncRNAs, including XLOC-085738 and XLOC-041531, are associated with steatosis-to-NASH progression. Collectively, our data provide a valuable resource into the molecular characterization of NASH progression, leading to the novel insight that GDF3 may be a potential noninvasive diagnostic biomarker for NASH while further showing that ferroptosis is a therapeutic target for the disease.

## Introduction

Due to lifestyle changes in modern society, the global prevalence of nonalcoholic fatty liver disease (NAFLD) is estimated to be >25% and will continue to rise [1–3]. NAFLD comprises a spectrum of liver disorders ranging from simple steatosis (NAFL) to nonalcoholic steatohepatitis (NASH). While NAFL is generally considered to be a benign condition, NASH is typically characterized by persistent liver injury and chronic inflammation, and it is prone to develop into severe end-stage liver diseases [4–7].

Currently, there are at least two major hurdles in the development and testing of NASH-related therapies. First, the gold standard to diagnose NASH is histopathological assessment based on liver biopsy, which has high risks of bleeding, infection, hepatic rupture, and even mortality [8]. Additionally, histopathological analysis is susceptible to sampling error and pathologist variability [8]. Establishment of reliable noninvasive circulating biomarkers is therefore an urgent clinical need to efficiently and safely diagnose NASH [8]. Second, there is no US Food and Drug Administration-approved pharmacological therapies for the treatment of NASH due to an incomplete understanding of the molecular mechanisms leading to its pathogenesis [4, 9]. Although a number of studies have posited a multiple-hit hypothesis involving lipotoxicity, activation of the hepatocyte inflammasome, and alterations of gut microbiota [4, 6, 10], there is still a lack of systematic insight into the molecular features of the progression from steatosis to NASH. In particular, focusing on a single-isolated molecule or signaling pathway may be inadequate to depict the full spectrum of the disease. Thus, a comprehensive understanding of molecular alterations that underlie NASH progression is urgently needed.

In recent years, multi-omics approaches have been applied to investigate the mechanisms of many human diseases [11–13]. For instance, RNA-sequencing (RNA-seq) has been used to interrogate global gene expression changes at the transcriptional level [14, 15], while competing endogenous RNA (ceRNA) crosstalk provides information on gene regulatory networks [16, 17]. Furthermore, mass spectrometry (MS)-based proteomics allows for large-scale protein characterization [18, 19]. Therefore, tracking disease information at multiple levels can bring novel insights into noninvasive diagnosis, molecular mechanisms, and therapeutic implications. Previously, transcriptomic [20–22], proteomic [23, 24], or plasma metabolomic analyses have been independently performed using samples from patients with NAFLD and from mouse models [25, 26]. However, these studies were performed in a single stationary NAFL or NASH state, which makes it difficult to determine at which stage the altered molecular pathways play a role. Indeed, to the best of our knowledge, there is no prior research reporting a comprehensive multi-omics analysis to identify the molecular characterization of steatosis-to-NASH progression.

A high-fat, high-cholesterol and high-fructose (HFHC) diet, also known as the Gubra Amylin NASH diet, has been used to induce liver pathology closely resembling human NASH in mice [27]. In our recent study, 8-week-old C57BL/6J male mice were divided into two groups, with the first group of mice fed a normal chow diet (ND) or HFHC diet for 12 weeks and the second group of mice fed an ND or HFHC diet for 28 weeks [28]. By examination of their metabolic phenotypes, liver pathology, and gene expression, we showed that the mice fed a HFHC diet for 12 weeks developed NAFL without obvious hepatic inflammation and injury, whereas the mice fed a HFHC diet for 28 weeks exhibited typical NASH, including pronounced liver inflammation and damage [28]. In the current study, we performed a comprehensive multi-omics analysis to investigate the alterations of mRNAs, miRNAs, lncRNAs, and proteins in the liver, as well as plasma metabolites, in NAFL and NASH mice using the models described above. Furthermore, we identified altered molecules, pathways, and metabolites involved in the steatosis-to-NASH progression through comparisons of these two groups of datasets. Based on these findings, a role for Growth Differentiation Factor 3 (GDF3) and ferroptosis in this progression was identified and thus their potential clinical significance and pathophysiological functions were investigated. In addition, several miRNAs, including miR-582-5p and miR-292a-3p, as well as lncRNAs, notably XLOC-085738 and XLOC-041531, were found to be associated with steatosis-to-NASH progression.

## Results

### Transcriptomic profiling associated with steatosis-to-NASH progression

**Figure 1:**
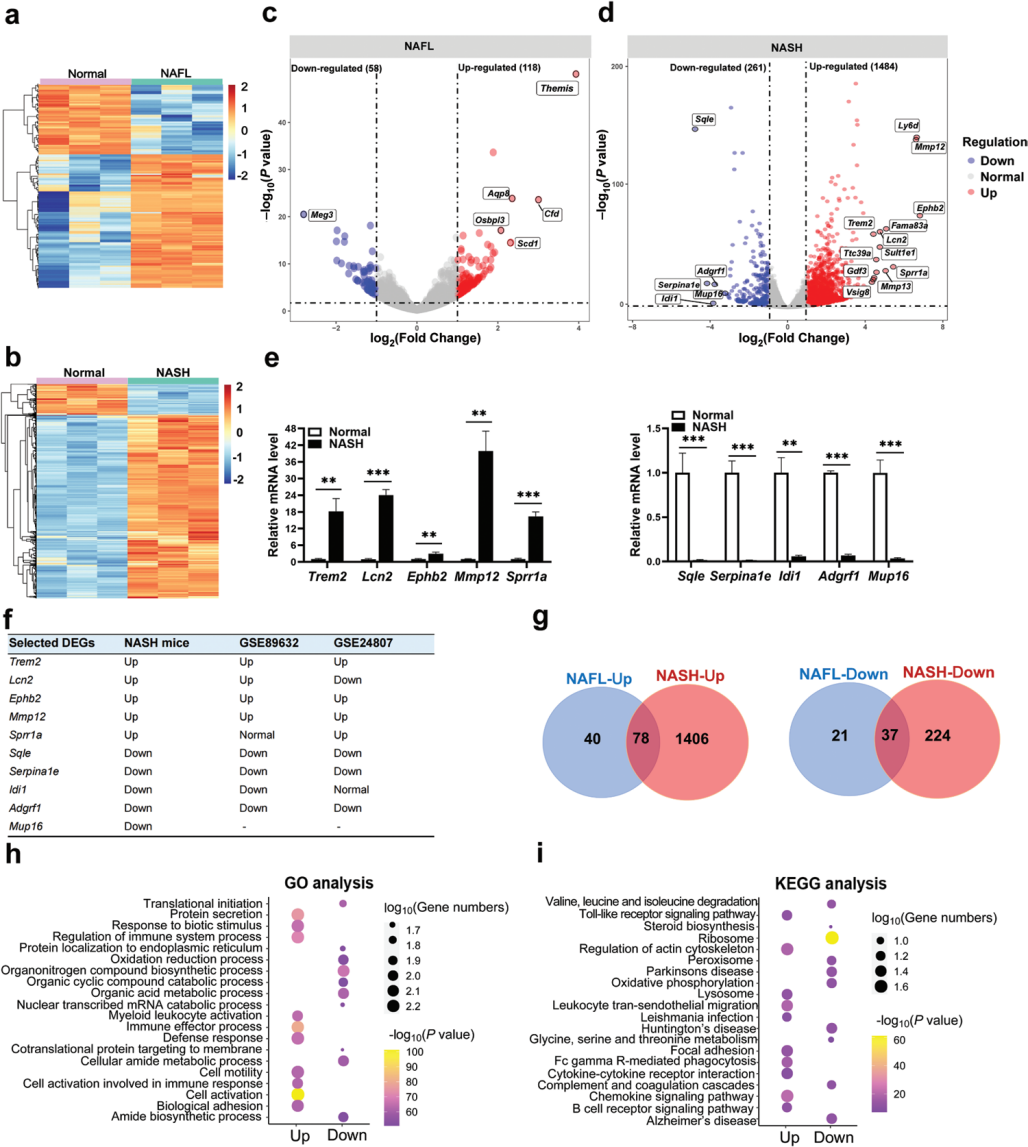
Transcriptomic profiling associated with steatosis−to−NASH progression. (a−f) DEGs in the livers of NAFL and NASH mice. (a and b) Heatmap showing the expression patterns of DEGs in the livers of NAFL (a) and NASH mice (b) compared with their age-matched normal controls after batch-effect correction, respectively. (c and d) Volcano plot of DEGs in the livers of NAFL (c) and NASH mice (d). The top dysregulated mRNAs are labeled as indicated. (e) mRNA expression levels of top 5 up-regulated and 5 down-regulated genes in NASH mice analyzed by qRT-PCR. (f) Validation of DEGs in two public datasets of NASH patients (GSE89632 and GSE24807). (g) Comparison of up-regulated (left) or down-regulated (right) genes in the livers of NAFL and NASH mice. (h and i) GO (h) and KEGG (i) analysis of NASH-specific genes. Data are represented as mean ± SEM. Two-tailed Student’s *t*-test was used in (e). Wald test by Deseq 2 was used in (f). ^**^P < 0.01, ^***^P < 0.001.

To obtain a comprehensive molecular understanding of steatosis-to-NASH progression, we compared NAFL and NASH mice with their age-matched healthy controls, respectively (Supplementary Fig. S1). We performed RNA-seq to analyze hepatic genome-wide mRNA expression patterns and generated gene expression density plots (Supplementary Fig. S2a and b). By correlation analysis, we demonstrated that gene expression reads correlated well among different samples (Supplementary Fig. S2c and d). Principal component analysis (PCA) showed that samples in each diet subgroup were close, while samples between NAFL and NASH mice with their matched normal controls were separated (Supplementary Fig. S2e and f). Hierarchical clustering analysis also showed that samples in each group clustered together (Fig. 1a and b). These results indicate that the dataset was reproducible and reliable for our downstream analysis.

Then, using a false discovery rate (FDR) < 0.01 and fold change > 2.0 as the cutoff threshold, we carried out differentially expressed genes (DEGs) analysis. We identified 176 DEGs in the livers of NAFL mice compared to age-matched normal control mice, of which 118 were up-regulated and 58 were down-regulated (Fig. 1c). The top 5 up-regulated genes in NAFL mice included the genes encoding for Themis, Cfd, Aqp8, Osbpl3, and Scd1 (Fig. 1c). In addition, we identified 1745 DEGs in NASH mice, of which 1484 were up-regulated and 261 were down-regulated (Fig. 1d). The top 5 up-regulated genes in NASH mice included those encoding for Trem2, Lcn2, Ephb2, Mmp12, and Sprr1a, while the top 5 down-regulated genes were those encoding for Sqle, Serpina1e, Idi1, Adgrf1, and Mup16 (Fig. 1d). We confirmed the expression changes of these 10 genes by quantitative real-time PCR (qRT-PCR) analysis (Fig. 1e). For the most part, similar changes in the direction of expression of these genes were observed in patients with NASH from two public RNA-Seq datasets (NCBI GEO, accession number: GSE89632 and GSE24807) (Fig. 1f).

To identify the molecular alterations underlying steatosis-to-NASH progression, we compared two sets of DEGs from NAFL and NASH mice. We found that 115 DEGs were identified in both NAFL and NASH mice, of which 78 genes were up-regulated and 37 down-regulated (Fig. 1g). Importantly, we found a total of 1630 NASH-specific DEGs, of which 1406 genes were up-regulated and 224 genes down-regulated (Fig. 1g). Gene ontology (GO) analysis showed that genes specifically up-regulated in NASH were mainly enriched in biological processes, including biotic stimulus response, immune regulation, and myeloid leukocyte activation, while down-regulated genes were related to organonitrogen compound biosynthesis and organic acid metabolism (Fig. 1h). Kyoto Encyclopedia of Genes and Genomes (KEGG) pathway enrichment results revealed that up-regulated genes were mainly enriched in toll-like receptor signaling, regulation of actin cytoskeleton, leukocyte trans-endothelial migration, and chemokine signal pathway, as well as cytokine and cytokine receptor interaction, while down-regulated genes were mainly enriched in valine, leucine, and isoleucine degradation, as well as steroid biosynthesis and glycine, serine and threonine metabolism (Fig. 1i). All of the DEGs in NAFL or NASH and their comparisons are listed in Supplementary document 1.

### GDF3 is specifically elevated in NASH mice

**Figure 2:**
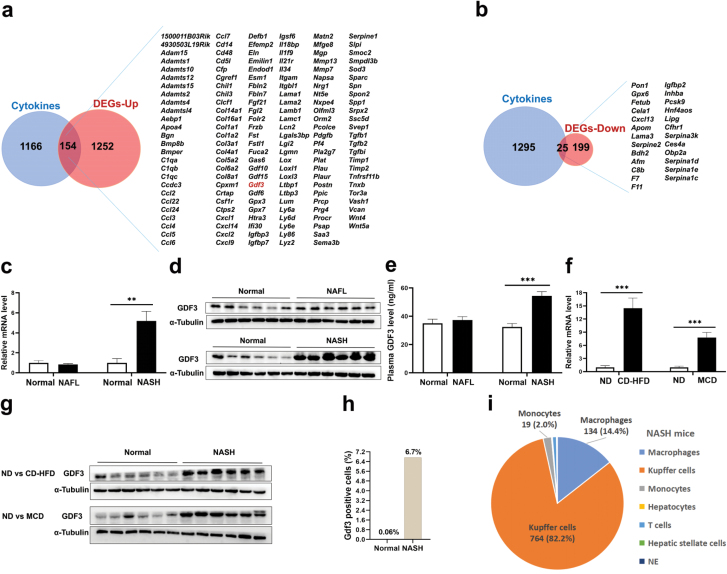
Gdf3 is specifically up-regulated in NASH mice. (a and b) Intersection of mRNAs specifically up-regulated (a) or down-regulated (b) in NASH mice with a mouse secretome database (NCBI GEO, accession number: GSE10246). (c) mRNA expression levels of *Gdf3* in the livers of NAFL mice (left) and NASH mice (right) with their age-matched normal controls, respectively. (d) Protein expression of GDF3 in the livers of NAFL mice (top) and NASH mice (bottom) with their matched normal controls. (e) Plasma GDF3 levels of NAFL mice (left) and NASH mice (right). (f) Relative mRNA levels of *Gdf3* in the livers of normal mice and NASH mice induced by CD-HFD (left) or MCD (right). In the CD-HFD experiment, C57BL/6J mice were fed a normal diet or CD-HFD for 10 weeks. In the MCD experiment, C57BL/6J mice were fed a normal diet or MCD for 6 weeks. (g) Protein expression of GDF3 in the livers of normal mice and NASH mice induced by CD-HFD (top) or MCD (bottom). (h) Proportion of cells expressing Gdf3 in the livers of normal mice or HFHC diet-induced NASH mice analyzed by single-cell sequencing. (i) The Gdf3-expressing cell types in the livers of NASH mice. Data are represented as mean ± SEM. ^**^P < 0.01, ^***^P < 0.001. Two-tailed Student’s *t*-test was used in (c, e, f). (c−g) *n* = 6 per group.

Of note, our GO analysis revealed that a cluster of NASH-specific DEGs were enriched in protein secretion (Fig. 1h). Indeed, recent studies have demonstrated that liver-secreted cytokines have an important role in many aspects of NAFLD-NASH development [29, 30]. To identify novel cytokines involved in steatosis-to-NASH progression, we overlapped 1630 NASH-specific DEGs with a mouse secretome database (GEO accession number: GSE10246). In this manner, we were able to identify 154 up-regulated cytokines and 25 down-regulated cytokines in livers from NASH mice (Fig. 2a and b). These included a range of well-established cytokines in NASH progression, including Ccl2, Cxcl1, and Tgfb1, as well as some recently identified NASH-related cytokines, including Bmp8b, Lcn2, Gdf10, and Gdf15 [31–34]. In addition, we noticed that GDF3, another member of the GDF family of proteins, was up-regulated in the livers of NASH mice (Fig. 2a). GDF3 was recently reported to be associated with obesity and systemic insulin resistance [35]. However, its expression and role in NASH pathogenesis has not been previously studied. Thus, GDF3 was chosen for further analysis.

We found that its mRNA and protein levels were significantly increased in the livers of NASH mice, but remained unchanged in NAFL mice (Fig. 2c and d). Consistent with these findings, plasma GDF3 concentrations were specifically increased in NASH mice (Fig. 2e). To determine whether the observed changes in GDF3 expression was an HFHC diet-specific effect, we examined GDF3 expression in two other NASH mouse models. In both choline-deficient high-fat-diet (CD-HFD) and methionine-choline-deficient diet (MCD)-induced NASH mice, we also observed a dramatic up-regulation of GDF3 in the livers compared to age-matched, normal diet-fed controls (Fig. 2f and g), suggesting that the overproduction of GDF3 is a conserved feature in NASH progression. We next analyzed GDF3 expression by a recent single-cell transcriptomic analysis [36], which confirmed the increased proportion of GDF3-positive cells in the livers of NASH mice (Fig. 2h). In addition, GDF3-positive cells were mainly identified as Kupffer cells (82.2%) and macrophages (14.4%) in NASH mice (Fig. 2i).

### GDF3 is a noninvasive diagnostic biomarker for NASH

**Figure 3:**
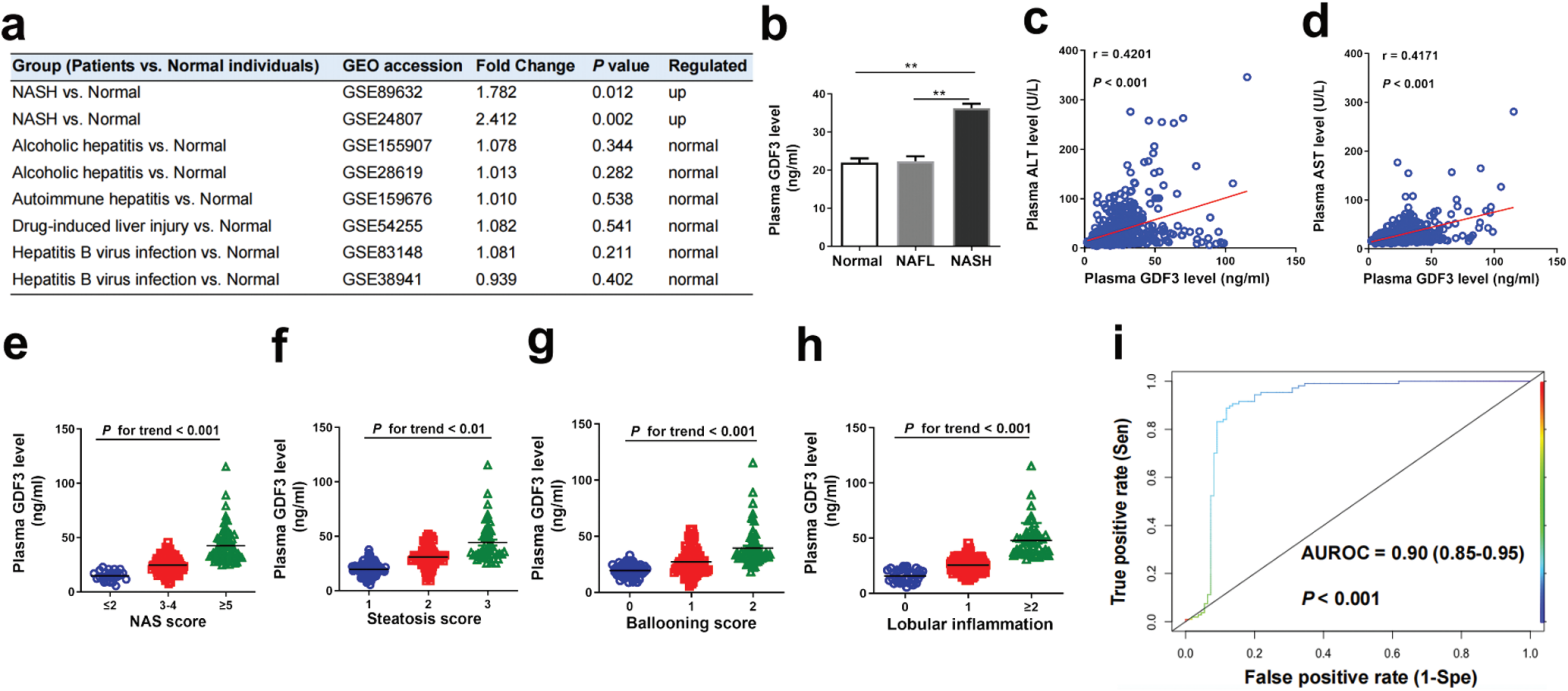
Plasma GDF3 concentration is elevated in patients with NASH. (a) Relative fold change of hepatic Gdf3 expression from public datasets of human individuals with different liver diseases compared to normal healthy individuals. (b−i) Analysis of plasma GDF3 concentrations in human subjects. Normal: *n* = 315, NAFL: *n* = 110, NASH: *n* = 107. (b) Comparison of plasma GDF3 levels in patients with NAFL or NASH compared to normal individuals. (c and d) The correlation between plasma GDF3 concentrations and ALT (c) and AST (d) in total participants. (e−h) Plasma GDF3 levels in patients with NAFL or NASH with different NASH score (e), steatosis score (f), ballooning score (g), and lobular inflammation (h). (i) NASH diagnostic accuracy in the total NAFLD cohort (*n* = 217). AUROC, area under the receiver-operating characteristic curve. Wald test by Deseq 2 was used in (a). One-way ANOVA with Tukey’s post hoc test was used in (b, e−h). Spearman correlation was used in (c, d). DeLong’s test was used in (i). ^**^P < 0.01, ^***^P < 0.001.

To understand the clinical relevance of our animal model-based observations, we analyzed GDF3 expression in the livers of patients with various liver diseases using GEO datasets. Interestingly, hepatic GDF3 expression was significantly increased in patients with NASH (Fig. 3a), but it was unchanged in patients with alcoholic hepatitis, autoimmune hepatitis, drug-induced liver injury, and hepatitis B virus infection (Fig. 3a). Next, we measured plasma GDF3 levels in healthy individuals and in patients with liver biopsy-proven NAFL and NASH. Compared to the normal healthy individuals and those with NAFL, plasma GDF3 levels were substantially higher in patients with NASH (Fig. 3b). Plasma GDF3 levels were positively correlated with alanine transaminase (ALT) and aspartate transaminase (AST) levels in all individuals (Fig. 3c and d). In addition, plasma GDF3 levels were gradually elevated with the NAFLD activity score (NAS) (Fig. 3e) and with the scores of steatosis and ballooning, as well as the degree of lobular inflammation (Fig. 3f−h). Furthermore, we constructed the area under a receiver-operating characteristic curve (AUROC) for plasma GDF3 concentrations and found that it exhibited a high accuracy for distinguishing patients with NASH from the total patients with NAFLD (AUROC = 0.90; 95% confidence interval: 0.85−0.95, *P* < 0.001) (Fig. 3i). At a cutoff point of 26.5 ng/mL, the sensitivity was 90.7% and the specificity was 86.4%, resulting in a positive predictive value of 86.6% and a negative predictive value of 90.5%. Collectively, these results highlight the potential utility of circulating GDF3 as a noninvasive diagnostic biomarker for NASH in patients with suspected NAFLD.

### Proteomic profiling of steatosis-to-NASH progression

**Figure 4:**
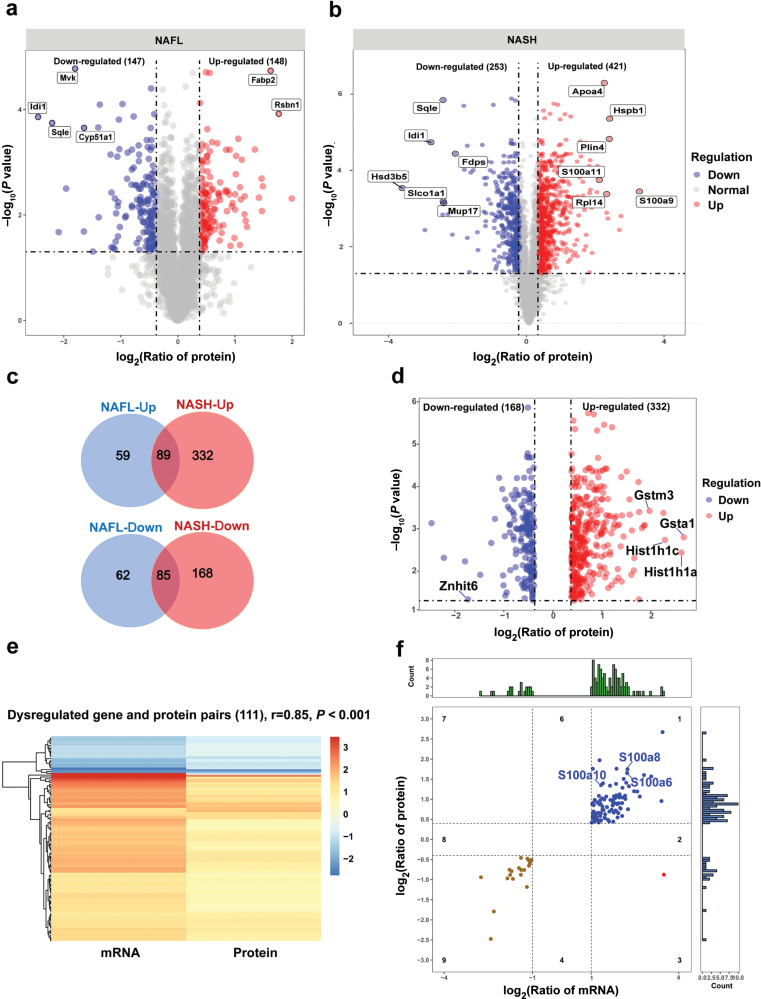
Proteomic profiling associated with steatosis-to-NASH progression. (a and b) Volcano plot of DEPs in the livers of NAFL (a) and NASH mice (b). The top DEPs are labeled as indicated. (c) Comparison of up-regulated (top) or down-regulated (bottom) proteins in the livers of NAFL and NASH mice. (d) Volcano plot of specifically altered proteins in NASH. (e and f) Integration of proteomic and transcriptomic profiles specifically altered in NASH. (e) Heatmap of differentially expressed genes and protein pairs. (f) Expression of gene-protein pairs. The blue circle dots represent up-regulated gene-protein pairs. The brown circle dots represent down-regulated gene-protein pairs. Spearman correlation was used in (e).

We carried out global proteomics of liver lysates from mice described above by MS analysis and identified 61,251 unique peptides that were mapped to 4800 proteins, of which 4242 were quantifiable. The reproducibility and reliability of the proteomic profile datasets were shown by correlation (Supplementary Fig. S3a and b) and PCA analysis (Supplementary Fig. S3c and d). In total, 295 differentially expressed proteins (DEPs) were identified in the livers of NAFL mice, of which 148 proteins were up-regulated and 147 were down-regulated (Fig. 4a). 674 DEPs were identified in the livers of NASH mice, of which 421 proteins were up-regulated and 253 were down-regulated (Fig. 4b).

Comparison of two groups of DEPs showed that 174 proteins (89 up-regulated and 85 down-regulated) were changed in both NAFL and NASH livers (Fig. 4c). In addition, 500 proteins were specifically altered in NASH mice (Fig. 4c), of which 332 proteins were up-regulated and 168 were down-regulated. The NASH-specific DEPs included several classes with distinct functions (Fig. 4d). For example, two proteins among the up-regulated ones were Gsta1 and Gstm3, which are both glutathione-S-transferases that could provide a protective role against intracellular stress, including lipid peroxidation (Fig. 4d). Also proteins among the up-regulated ones were Hist1h1a and Hist1h1c, which both belong to the H1-linker histone family, reflecting that changes in chromatin organization and remodeling occur during NASH progression (Fig. 4d). On the other hand, Znhit6 (also known as BCD1), which is an assembly factor required for the maintenance of box C/D small nucleolar RNAs (snoRNAs) levels [37], was identified among the down-regulated proteins (Fig. 4d). snoRNAs have been increasingly implicated in the modification of ribosomes and crucial for the control of cellular post-transcriptional processes [37]. All of DEPs in NAFL or NASH and their comparisons are presented in Supplementary document 2.

Joint analysis of the transcriptome and proteome for a given disease can provide a more accurate overview of the dynamic process involved in its progression [38]. We therefore integrated the proteomic and transcriptomic profiles of the proteins and genes specifically altered in NASH mice. This revealed that 111 gene and protein pairs were differentially expressed with high correlation coefficient (r = 0.85, Fig. 4e), of which 90 pairs were up-regulated and 21 down-regulated (Supplementary document 2). This relatively low number of transcript-protein pairs is consistent with previous transcriptome-proteome comparison studies in other contexts [39, 40]. Among the 90 pairs that were up-regulated, we identified S100A6, S100A8, and S100A10 (Fig. 4f). S100 proteins, consisting of ~20 members, are calcium-binding proteins and have garnered much attention due to their involvement in several human diseases [41]. Interestingly, our proteomic profiles showed that the protein expression levels of S100A9 and S100A11 were up-regulated in the livers of both NAFL and NASH mice (Supplementary document 2). These results demonstrate the involvement of S100A9 and S100A11 in the initiation of NAFL, while S100A6, S100A8, and S100A10 are involved in steatosis-to-NASH progression.

### Plasma metabolite analysis of steatosis-to-NASH progression

**Figure 5:**
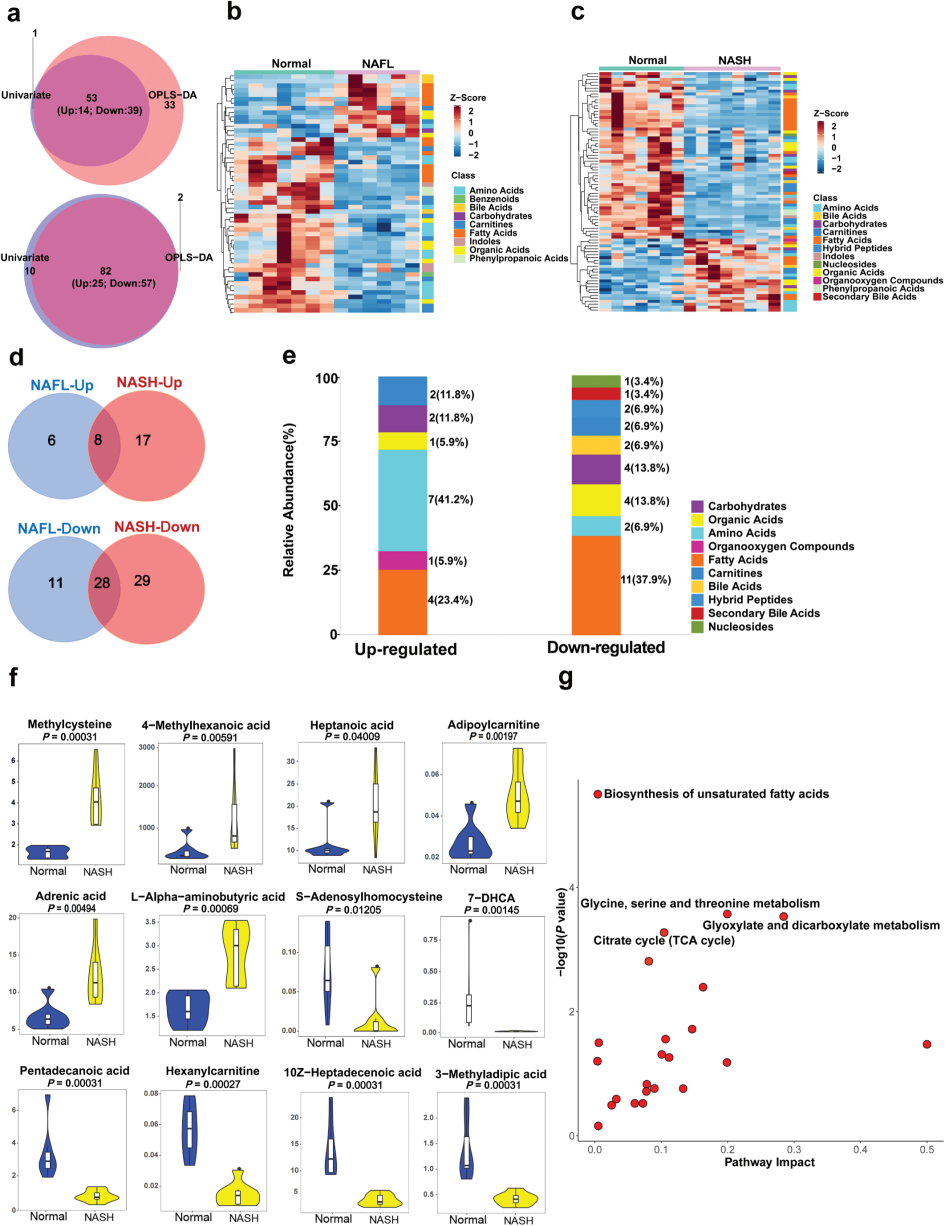
Plasma metabolomics analysis associated with steatosis-to-NASH progression. (a−d) Altered plasma metabolites of NAFL and NASH mice. (a) Intersection of dysregulated metabolites between Univariate analysis and OPLS-DA analysis of NAFL mice (top) and NASH mice (bottom). (b and c) Heatmap showing the patterns between dysregulated metabolites in NAFL mice (b) and NASH mice (c) after batch-effect correction. (d) Comparison of up-regulated (top) or down-regulated (bottom) metabolites in NAFL and NASH mice. (e−g) Analysis of specifically altered plasma metabolites in NASH mice. (e) The category of dysregulated metabolites. (f) Vioplot of top 5 increased or decreased plasma metabolites. (g) Functional enrichment pathway analysis of dysregulated metabolites.

Using liquid chromatograph-MS (LC-MS), we identified 210 plasma metabolites from mice described above. The reproducibility and reliability of the plasma metabolic profiles in NAFL and NASH mice were examined by PCA analysis (Supplementary Fig. S4a and b). Volcano plots of dysregulated metabolites in NAFL (Supplementary Fig. S4c and e) and NASH mice (Supplementary Fig. S4d and f) were first analyzed by Univariate analysis and Orthogonal Partial Least Squares Discrimination analysis (OPLS-DA), respectively. Then, through combination of these two analyses, we identified 53 altered plasma metabolites (14 up-regulated, 39 down-regulated) in NAFL mice and 82 dysregulated plasma metabolites (25 up-regulated, 57 down-regulated) in NASH mice (Fig. 5a). We then created a heatmap based on the normalized expression content of dysregulated metabolites, which indicated that samples in each group clustered closely, and the two groups had a different expression pattern (Fig. 5b and c). In addition, the content of these dysregulated metabolites within the two groups of mice correlated well (Supplementary Figs. S5 and S6).

Next, we compared the levels of plasma metabolites between NAFL and NASH mice and found that a total of 36 plasma metabolites (8 up-regulated, 28 down-regulated) were dysregulated in both NASH and NAFL mice (Fig. 5d). More importantly, 46 plasma metabolites were specifically altered in NASH mice (Fig. 5d), of which 17 metabolites were up-regulated and 29 down-regulated. Collectively, the most abundant metabolites specifically altered in the NASH mice included fatty acids (4 up-regulated and 11 down-regulated), followed by amino acids (7 up-regulated and 2 down-regulated) and then carbohydrates (2 up-regulated and 4 down-regulated) (Fig. 5e). Among all the specifically altered plasma metabolites in NASH mice, the levels of methylcysteine, 4-methylhexanoic acid, heptanoic acid, adipoylcarnitine, adrenic acid, and l-alpha-aminobutyric acid were increased, while S-adenosylhomocysteine, 7-dehydrocholic acid, pentadecanoic acid, hexanylcarnitine, 10Z-heptadecenoic acid, and 3-methyladipic acid were decreased (Fig. 5f). Functional enrichment pathway analysis showed that the specifically altered plasma metabolites in NASH were enriched in a spectrum of metabolic processes, including biosynthesis of unsaturated fatty acids and glycine, serine and threonine metabolism, as well as the tricarboxylic acid cycle (Fig. 5g). All of the altered plasma metabolites in NAFL or NASH and their comparisons are listed in Supplementary document 3.

### The integration of proteomic and metabolomic datasets

**Figure 6:**
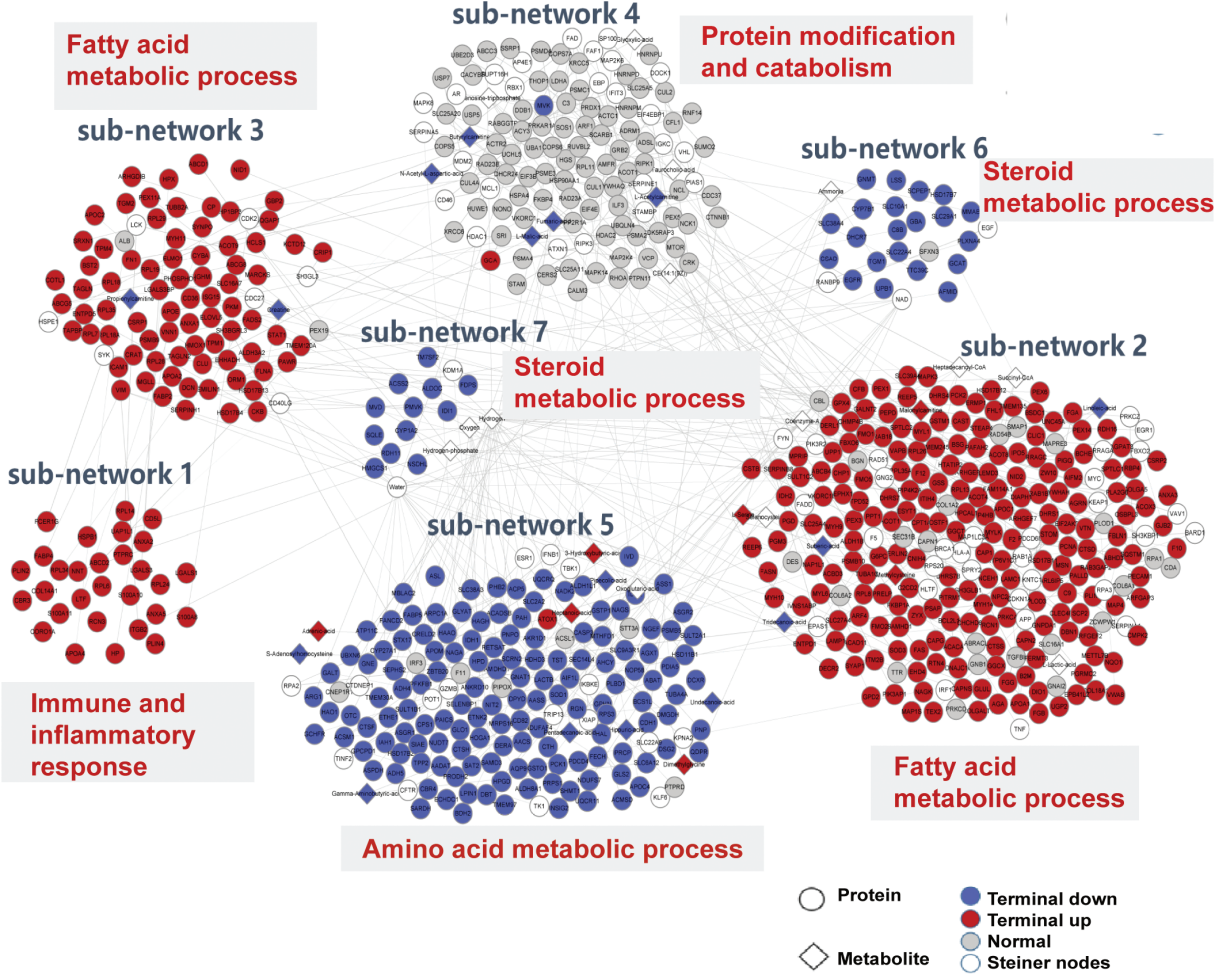
Protein and metabolite interaction network associated with steatosis-to-NASH progression. GO analysis results after integrating protein–protein and protein–metabolite interactomes into a network using the PCSF network model.

We further analyzed the interaction between specifically altered proteomic and metabolomic profiles in NASH mice. Using the network model Prize Collecting Steiner Forest (PCSF), we characterized the protein–protein and protein–metabolite interactome network. With the information presented as cluster-grams, we developed and re-arranged the whole network into seven sub-networks incorporating 617 proteins and 24 metabolites (Fig. 6). GO analysis showed that these sub-networks were enriched in immune and inflammatory response (sub-network 1), fatty acid metabolic process (sub-network 2 and 3), protein modification and catabolism (sub-network 4), amino acid metabolic process (sub-network 5), and steroid metabolic process (sub-network 6 and 7). Most of metabolites in the sub-networks were fatty acids, including linoleic-acid, pentadecanoic acid, and adrenic acid.

**Figure 7:**
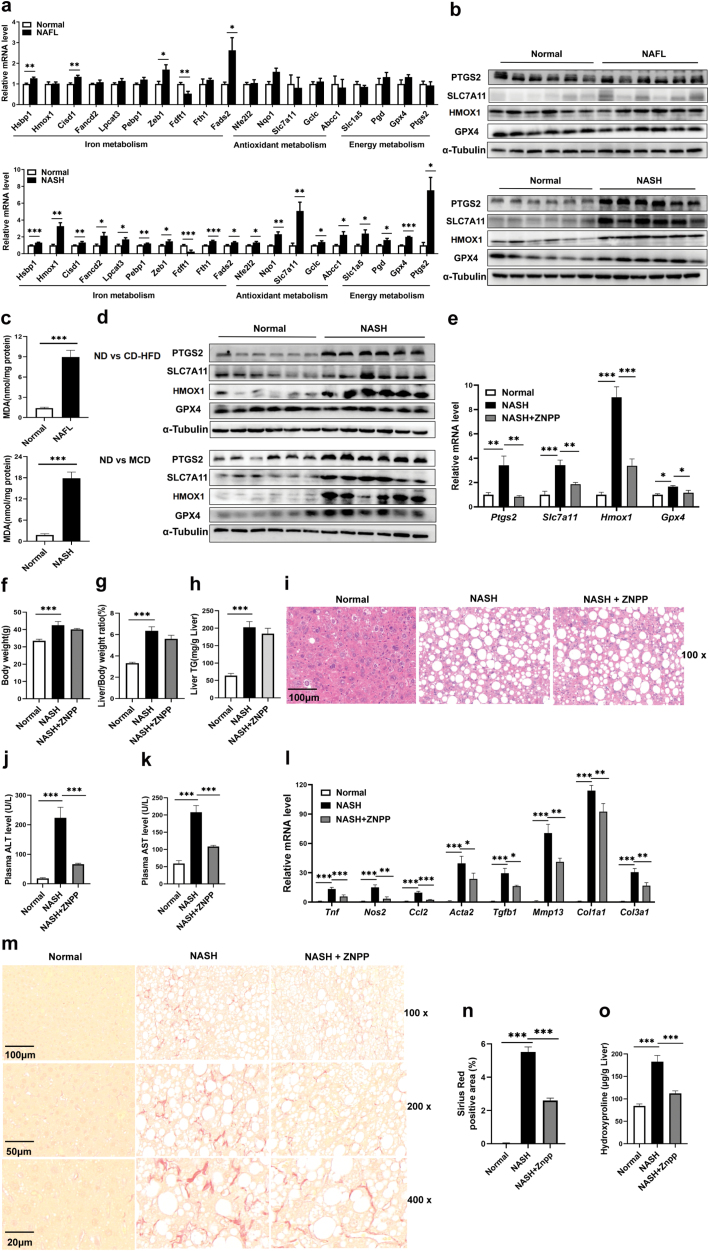
Pharmacological suppression of ferroptosis improves long-term HFHC diet-induced NASH in mice. (a) Relative mRNA levels of ferroptosis-related genes involved in iron metabolism, antioxidant metabolism, and energy metabolism in NAFL (top) and NASH mice (bottom). (b) Protein expression of ferroptosis markers in NAFL (top) and NASH (bottom) mice. (c) Hepatic MDA content was measured to determine lipid peroxidation in NAFL (top) and NASH (bottom) mice with their age-matched normal controls. (d) Protein expression of ferroptosis markers in normal mice or NASH mice induced by CD-HFD (up) and MCD (bottom). (e−o) 8-week-old male C57BL/6J mice were fed a normal diet or HFHC diet for 34 weeks. Then, normal mice were treated with vehicle control while NASH mice were administered ZnPP (10 mg/kg, i.p.) or vehicle control for another 4 weeks. (e) Relative mRNA levels of *Ptgs2*, *Slc7a11*, *Hmox1,* and *Gpx4* in the liver of three groups of mice. (f−h) Body weights (f), liver/body weight ratio (g), and hepatic triglyceride contents (h) among three groups of mice. (i) Hematoxylin and eosin (H&E) staining of liver sections. (j and k) Plasma ALT and AST levels. (l) Relative mRNA levels of genes involved in hepatic inflammation and fibrosis. (m) Representative Sirius red staining of liver sections using different magnifications. (n) Quantification of Sirius red staining images. (o) Hepatic hydroxyproline levels from three groups of mice. Data are represented as mean ± SEM. (a−o) *n* = 6 per group. Two-tailed Student’s *t*-test was used in (a and c). One-way ANOVA with Tukey’s post hoc test was used in (e−h, j−l, n, o). ^*^P < 0.05, ^**^P < 0.01, ^***^P < 0.001.

Interestingly, we found that proteins involved in sub-network 2, 3, and 5 included GPX4, ACACA, NQO1, GDPD, PGD, HMOX1, FADS, GSL2, FANCD, and SQLE (Supplementary Fig. S7a−c), which are associated with ferroptosis [42]. Consistent with these results, functional enrichment analysis confirmed that these sub-networks were related to oxidative stress, response to metal iron, and glutathione metabolism (Supplementary Fig. S7d). By qRT-PCR and western blotting, we confirmed that mRNA and protein expression levels of ferroptosis-related genes and their proteins, including Hmox1, Slc7a11, Ptgs2, and Gpx4, remained unaltered or only slightly changed in the livers of NAFL mice, but were significantly up-regulated in NASH mice (Fig. 7a and b). Among these gene products, Slc7a11 and Gpx4 are protective regulators, while Hmox1 and Ptgs2 are biomarkers of ferroptosis. In addition, more severe lipid peroxidation was detected in the livers of NASH mice compared with NAFL mice, as shown by higher hepatic malondialdehyde (MDA) content in the former group of mice (Fig. 7c). Thus, the up-regulation of Slc7a11 and Gpx4 in the livers of NASH mice might be a compensatory response and thus a self-protection mechanism to alleviate lipid peroxidation.

Up-regulation of Ptgs2, Slc7a11, Hmox1, and Gpx4 was also observed in CD-HFD- and MCD-fed mice (Fig. 7d), suggesting that ferroptosis is a common feature in NASH. We therefore explored the significance of ferroptosis. HFHC diet-induced NASH mice were treated with zinc protoporphyrin IX (ZnPP), a competitive Hmox1 inhibitor. As a result, up-regulation of Ptgs2, Slc7a11, Hmox1, and Gpx4 in the livers of NASH mice were significantly ameliorated by ZnPP treatment (Fig. 7e). While body weights, the liver/body weight ratio, and hepatic triglyceride content were not affected (Fig. 7f–i), plasma ALT and AST levels were markedly reduced in the NASH mice treated with ZnPP compared to vehicle control-treated NASH mice (Fig. 7j and k). The expression levels of several genes related to hepatic inflammation and liver fibrosis in NASH mice were also significantly down-regulated by ZnPP treatment compared to vehicle control-treated NASH mice (Fig. 7l). The improvement of liver fibrosis in the ZnPP-treated NASH mice was confirmed by hepatic Sirius red staining (Fig. 7m and n) and by a reduction of hepatic hydroxyproline contents (Fig. 7o). Our results demonstrated that liver fibrosis could be significantly improved within 4 weeks of interventions in mice, which is also observed in previous studies [43, 44]. One possible explanation is that diet-induced liver fibrosis might be mild. Indeed, it has been reported that diet-based models show a lack of full progression to severe fibrosis, even after long-term feeding [45, 46], and thus the mild symptoms of liver fibrosis in NASH mice might be relatively easy to alleviate. Even so, collectively, our findings indicate that pharmacological suppression of Hmox1 may be a therapeutic approach in the treatment of NASH.

### ceRNAs network analysis of steatosis-to-NASH progression

**Figure 8:**
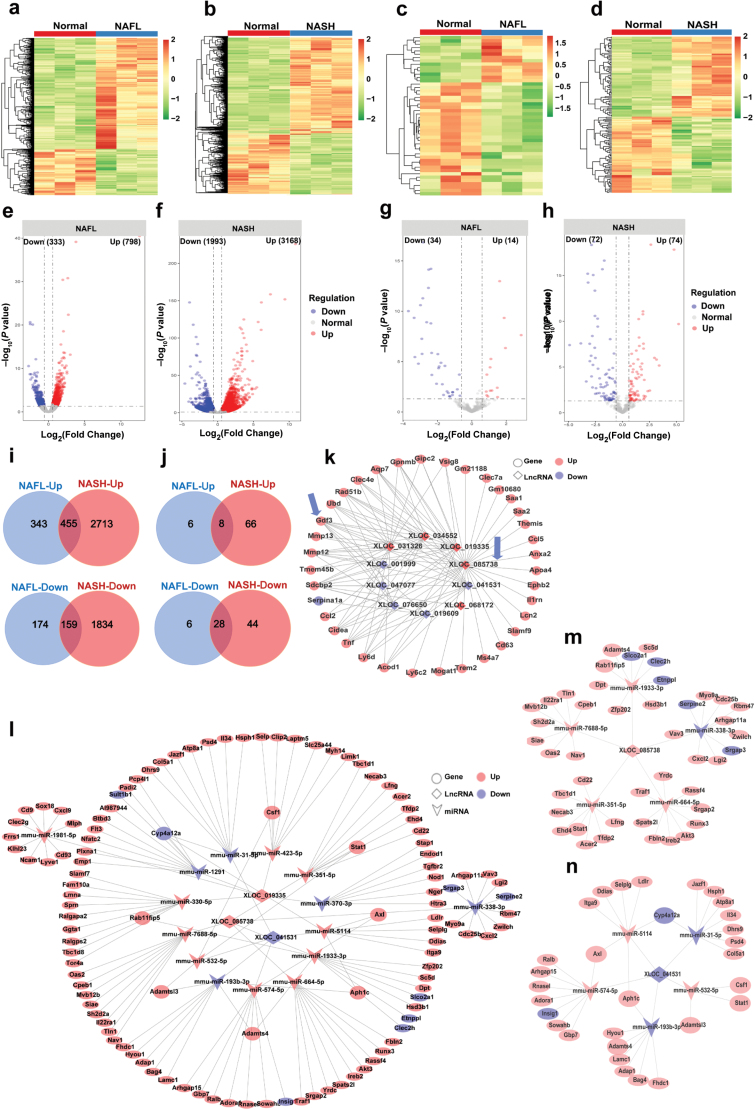
Non-coding RNA profiling associated with steatosis-to-NASH progression. (a−j) Differentially expressed non-coding RNAs in the livers of NAFL and NASH mice. (a and b) Heatmap showing the expression patterns of lncRNAs in NAFL mice (a) and NASH mice (b) with their matched normal controls after batch-effect correction. (c and d) Heatmap showing the expression patterns of miRNAs in NAFL mice (c) and NASH mice (d) with their normal controls after batch-effect correction. (e and f) Volcano plot of dysregulated lncRNAs in NAFL mice (e) and NASH mice (f). (g and h) Volcano plot of dysregulated miRNAs in NAFL mice (g) and NASH mice (h). (i) Comparison of up-regulated (top) or down-regulated (bottom) lncRNAs in NAFL and NASH mice. (j) Comparison of up-regulated (top) or down-regulated (bottom) miRNAs in NAFL and NASH mice. (k) CNC network analysis in NASH. The interaction between dysregulated lncRNAs and mRNAs in NASH was integrated into a network. (l−n) ceRNA network of differentially expressed lncRNAs, differentially expressed miRNAs and DEGs in NASH. (l) The interactions between differentially expressed lncRNAs and miRNAs, as well as related DEGs, are integrated into a regulation network. (m) Sub-network in the ceRNA network of lncRNA XLOC-085738, differentially expressed miRNAs and DEGs. (n) Sub-network in the ceRNA network of lncRNA XLOC-041531, differentially expressed miRNAs and DEGs.

Growing evidence suggests that non-coding RNAs might be crucial regulators in chronic liver diseases [47]. Overall, 50,177 lncRNAs and 420 miRNAs were detected by lncRNA sequencing and miRNA sequencing, respectively. We also determined the lncRNA expression density in the livers of NAFL and NASH mice (Supplementary Fig. S8a and b). The reproducibility and reliability of lncRNA profile datasets in NAFL and NASH mice were demonstrated by correlation analysis (Supplementary Fig. S8c and d) and hierarchical clustering analysis (Fig. 8a and b).

The miRNA expression density in the livers of NAFL and NASH mice was also determined (Supplementary Fig. S8e and f). The reproducibility and reliability of miRNA profile datasets in NAFL and NASH mice were demonstrated by correlation analysis (Supplementary Fig. S8g and h) and hierarchical clustering analysis (Fig. 8c and d). Compared to control mice, a total of 1131 lncRNAs (798 up-regulated and 333 down-regulated) and 5161 lncRNAs (3168 up-regulated and 1993 down-regulated) were differentially expressed in livers of NAFL and NASH mice, respectively (Fig. 8e and f). A total of 48 miRNAs (14 up-regulated and 34 down-regulated) and 146 miRNAs (74 up-regulated and 72 down-regulated) were differentially expressed in livers of NAFL and NASH mice, respectively (Fig. 8g and h).

Next, comparisons of up-regulated or down-regulated lncRNAs and miRNAs of NAFL and NASH mice were performed (Fig. 8i and j). 614 lncRNAs (455 up-regulated, 159 down-regulated) were differentially expressed in both NASH and NAFL mice (Fig. 8i), while 4547 lncRNAs (2713 up-regulated, 1834 down-regulated) were specifically altered in NASH mice (Fig. 8i). 36 miRNAs (8 up-regulated, 28 down-regulated) were differentially expressed in both NASH and NAFL mice (Fig. 8j), while 110 miRNAs (66 up-regulated, 44 down-regulated) were specifically altered in NASH mice (Fig. 8j). The top 5 specifically up-regulated expressed lncRNAs in NASH mice were XLOC_019582, XLOC_018429, XLOC_070784, XLOC_088151, and XLOC_032108, while the top 5 down-regulated lncRNAs were XLOC_058374, XLOC_013532, XLOC_099594, XLOC_028152, and XLOC_063066. The top 5 specifically up-regulated miRNAs in NASH were miR-582-5p, miR-582-3p, miR-342-5p, miR-155-5p, and miR-212-5p, while the top 5 down-regulated miRNAs were miR-292a-3p, miR-219a-2-3p, miR-376c-3p, miR-291a-3p, and miR-370-3p. The detailed information of these differentially expressed lncRNAs and miRNAs and their comparisons are listed in Supplementary document 4 and 5.

Next, we focused on the NASH-specific lncRNAs to understand their role in steatosis-to-NASH progression. Coding and non-coding gene co-expression (CNC) network was established using co-expressed lncRNA-mRNA to predict the target genes, as well as potential functions of lncRNAs. Ten lncRNAs and related DEGs were chosen for CNC network analysis, which enabled us to identify potential correlated mRNAs (Fig. 8k). In the CNC network, each lncRNA can be associated with dozens of mRNAs. As a result, the up-regulated lncRNA XLOC-085738 was implicated to be positively correlated with DEGs, including Lcn2, Trem2, Cd63, and II1rn (Fig. 8k), all of which are associated with NASH progression. Together, our results demonstrate the potential roles of lncRNAs in regulating steatosis-to-NASH progression.

ceRNAs can affect gene expression at the post-transcriptional level [16, 17]. Thus, we explored the potential role of non-coding RNAs acting as ceRNAs in NASH. Three dysregulated lncRNAs, 13 dysregulated miRNAs and related DEGs were included to construct a ceRNA network (Fig. 8l). We found that these lncRNAs and mRNAs can function as ceRNAs to compete for a common miRNA. The NASH-specific up-regulated lncRNA XLOC-085738 was indicated in the analysis to bind with miR-1933-3p, miR-664-5p, miR-351-5p, and miR-7688-5p to affect the expression of target mRNAs, including Cxcl2, Serpine2, and Cd22 (Fig. 8m). Some lncRNAs can interact with multiple miRNAs thereby affecting their binding to target mRNAs. For instance, the NASH-specific down-regulated lncRNA XLOC-041531 can bind with miR-5114 or miR-574-5p to regulate Axl; bind with miR-5114 or miR-193b-3p to regulate Aph1c; and bind with miR-532-5p or miR-193b-3p to regulate Adamtsl3 (Fig. 8n). Collectively, our findings indicate that lncRNA XLOC-085738 and XLOC-041531 may exert important roles in hepatic dysfunction that occurs during steatosis-to-NASH progression.

Moreover, by combining CNC and ceRNA analysis in NASH, 116 common mRNAs were predicted. Among them, 83 mRNAs, including Cxcl2, Cxcl13, Cxcl9, Slc7a11, Postn, Il1rn, CD53, CD33, CD5l, and CD9, had been identified in the transcriptomic analysis as the DEGs (Supplementary Table S1). GO analysis found that those genes were enriched in regulation of wound healing, external stimuli response, regulation of inflammatory response, myeloid leukocyte migration, and cytokine mediated signaling pathway (Supplementary Fig. S9a). KEGG pathway results showed that they were mainly related to osteoclast differentiation, complement and coagulation cascade, NF-κB signaling pathway and cytokine–cytokine receptor interactions (Supplementary Fig. S9b).

## Discussion

In the present study, using two periods of HFHC diet-feeding, we performed a large-scale integrative analysis of liver tissues from NAFL and NASH mice with their age-matched normal controls. Overall, our multi-omics study captured 176 mRNAs, 1131 lncRNAs, 48 miRNAs, 295 proteins, and 53 plasma metabolites that were altered in NAFL mice compared to age-matched normal mice. Meanwhile, 1745 mRNAs, 5161 lncRNAs, 146 miRNAs, 674 proteins, and 82 plasma metabolites were altered in NASH mice compared to age-matched normal mice. Through comparisons of these alterations in two mouse models, a total of 1630 mRNAs, 4547 lncRNAs, 110 miRNAs, 500 proteins, and 46 plasma metabolites were specifically altered in NASH mice compared to NAFL mice. Importantly, the different datasets showed a good correlation among each other. Several novel molecular features were demonstrated through the integration of our multi-dimensional datasets. Networks integrating protein–protein and protein–metabolite interactions revealed the potential role of ferroptosis in NASH. CNC analysis and ceRNA network showed that lncRNA XLOC-085738 and lncRNA XLOC-041531 may be associated with steatosis-to-NASH progression by regulating the expression of multiple mRNAs. Therefore, in addition to uncovering the molecular features of steatosis-to-NASH progression, our studies also identified a novel biomarker for NASH diagnosis, as well as confirming that ferroptosis is a potential therapeutic target to treat NASH.

Transcriptional profiling analysis revealed that the up-regulated NASH-specific genes were mainly enriched in immune and inflammatory response, including leukocyte activation and metastasis, and immune response. Lobular inflammation is considered one of the driving factors in NASH progression [48]. Both innate immunity and lymphocyte-mediated acquired immunity can trigger liver inflammation to promote NASH progression [10]. In addition, a myriad of dysregulated genes that are known to promote NASH were identified here, including Lcn2, Il1ra, and Postn. Lcn2 mediates NASH by promoting neutrophil-macrophage crosstalk via the induction of CXCR2 [49]. Circulating Il1ra concentrations are associated with liver inflammation and ALT levels [50]. Our previous study, as well as other findings, showed that periostin, encoded by *Postn*, could promote hepatic steatosis, inflammation, and fibrosis [51, 52]. More importantly, through transcriptomic analysis, we found that GDF3 expression is specifically up-regulated in the livers of NASH mice, while remaining unaltered in NAFL mice. We noticed that GDF3 was not identified in the proteomic analysis, which might be attributed to the fact that MS is not as sensitive as transcriptomic screening. Indeed, a recent study demonstrated that the protein secretome could not be accurately reflected by measuring changes in the transcriptome obtained from the same biological sample [40]. Therefore, we validated the elevated protein levels of GDF3 by western blot in multiple NASH mouse models and obtained consistent results. In agreement, plasma GDF3 concentrations were markedly increased in patients with NASH compared to patients with NAFL or healthy individuals. Plasma GDF3 levels were strongly associated with the NAS score and individual histologic features, including steatosis, ballooning, and lobular inflammation. We further evaluated the diagnostic potential of circulating GDF3, which demonstrated that it could be a noninvasive diagnostic biomarker for NASH with high accuracy (AUROC = 0.90). Moreover, integration of single-cell analysis found that elevated GDF3 is mainly derived from Kupffer cells and macrophages, which further supports the notion that macrophage-derived cytokines are potential good diagnostic markers of NASH [53].

Our plasma metabolome and comparison analysis showed that fatty acids, as well as amino acids, carbohydrates, and organic acids, were specifically changed in NASH. Through interaction sub-networks between dysregulated proteins and metabolites, abnormal expression of ferroptosis-associated proteins, including PTGS2, SLC7A11, HMOX1, and GPX4, was identified. Subsequent functional *in vivo* studies indicated that pharmacological suppression of HMOX1 can alleviate liver injury, inflammation, and fibrosis in long-term HFHC diet-induced NASH mice. Thus, activation of ferroptosis might be a potential mechanism that induces hepatocyte death in NASH progression. Ferroptosis is a recently recognized form of iron-dependent cell death induced by excessive lipid peroxidation and reactive oxygen species. It can play important pathophysiologic roles in the development of several chronic diseases, including tumorigenesis, cardiovascular diseases, and neurodegenerative diseases [42, 54]. While apoptosis has been well-studied as the most common type of hepatocyte death in the spectrum of NAFLD progression [55], it is a physiological process that also occurs in normal cells [56]. However, our results showed that ferroptosis-related genes were specifically altered in the livers of NASH mice, but was comparable between NAFL and normal mice. Therefore, suppression of ferroptosis might be a reliable strategy for treating NASH or its progression.

It has been well-established that miRNAs can bind target mRNAs to regulate gene expression through inhibiting mRNA translation or initiating degradation, and lncRNAs can bind miRNAs to competitively prevent the interaction between miRNAs and mRNAs [57]. In order to achieve a comprehensive understanding of ceRNA regulatory mechanisms in steatosis-to-NASH progression, CNC analysis was performed with differentially expressed non-coding RNAs and DEGs. We found that the up-regulated lncRNA XLOC-085738 can positively regulate the expression of many NASH-related genes. Furthermore, we developed a gene regulatory network by integrating the co-expression patterns of miRNA-mRNA, miRNA-lncRNA, and lncRNA-mRNA. We found that the down-regulated lncRNA XLOC-041531 can competitively bind multiple dysregulated miRNAs to affect the expression of a common mRNA. Therefore, our results identified the lncRNAs XLOC-085738 and XLOC-041531 as important regulators involved in steatosis-to-NASH progression. Moreover, by combining CNC and ceRNA analysis, 118 common mRNAs were identified, including chemokine family (such as Cxcl2, Cxcl13, and Cxcl9), transmembrane 4 superfamily (such as CD9 and CD53) and cytokines (such as Postn and Il1rn). Animal studies and clinical observations have confirmed that several members of the chemokine family are involved in the occurrence and development of metabolic liver diseases [58, 59]. In addition, members of four transmembrane protein superfamilies can regulate cell growth, migration, and adhesion [60], suggesting that their abnormal expression might affect the migration of liver immune and stellate cells, to influence hepatic inflammation and fibrosis.

There are several limitations to our study. First, we should point out that the multi-omics data were derived from the lysates of whole livers, as some technologies like proteomic and metabonomic analysis have yet to be well-developed on the single-cell level. Second, most of our findings were based on mouse models. The translatability of our results from animal models to humans need further studies. Thirdly, mechanistic studies using GDF3 knockout and/or transgenic mice, especially cell- or tissue-specific manipulations, would be required to further evaluate the role of GDF3 in NASH progression.

Taken together, to the best of our knowledge, this is the first study to explore the molecular characterization of steatosis-to-NASH progression through a large-scale system biology approach. We also integrated multiple types of omic datasets into a network that identified several novel potential genes, proteins, metabolites, and non-coding RNAs associated with steatosis-to-NASH progression, which will provide a valuable resource to explore the molecular mechanisms of NASH progression. Moreover, our findings identified GDF3 and ferroptosis as a potential diagnostic and therapeutic target, respectively, for NASH.

## Materials and methods

### Animal experiments

Male C57BL/6J mice aged 6 weeks were purchased from Shanghai Laboratory Animal Company (SLAC, Shanghai, China). After 2 weeks of acclimatization, mice were fed a ND or HFHC diet (D09100310, Research Diets Inc, New Brunswick, USA) for 2 different periods as described previously [28]. In the NAFL group, mice were fed a ND or HFHC diet for 12 weeks. In the NASH group, mice were fed a ND or HFHC diet for 28 weeks. Mice were anesthetized with sodium-pentobarbital (Nembutal, 80 mg/kg, i.p.) and killed after a 6 h fast. Blood samples and tissues were harvested for further analysis. For CD-HFD feeding, C57BL/6J mice were fed a CD-HFD (A06071302, Research Diets Inc, New Brunswick, USA) for 10 weeks. For MCD feeding, C57BL/6J mice were fed a methionine and choline-deficient diet (A02082002BR, Research Diets Inc, New Brunswick, USA) for 6 weeks. For ZnPP treatment, mice were fed a ND or HFHC diet for 34 weeks followed by daily intraperitoneal injection of ZnPP (15442-64-5, MedChemExpress, 10 mg/kg) or vehicle control for another 4 weeks. All mice were housed at 21 ± 1°C with a humidity of 55% ± 10% and 12-h light/12-h dark cycle in a specific-pathogen-free facility and had free access to water and food.

### RNA-seq and data processing

A total amount of 3 µg of RNA per sample was used as input material for the RNA sample preparations. Sequencing libraries were generated using NEBNext® Ultra™ RNA Library Prep Kit for Illumina® (NEB, USA) following the manufacturer’s recommendations, and index codes were added to attribute sequences to each sample. Library quality was assessed on the Agilent Bioanalyzer 2100 system. The sequencing libraries were sequenced on an Illumina Hiseq2500/X platform. Index of the reference genome was built using Bowtie v2.2.3 and paired-end clean reads were aligned to the reference genome using TopHat v2.0.12. Cuffquant and cuffnorm (v2.2.1) was used to calculate FPKMs of genes in each sample. Gene FPKMs were computed by summing the FPKMs of transcripts in each gene group. Differential expression analysis of two groups was performed using the DESeq2 R-package. For RNA-Seq, an adjusted FDR < 0.01 and |Fold Change| ≥ 2 was used as the cutoff values for identifying DEGs. For lncRNA-Seq and miRNA-Seq, the cutoff was FDR < 0.05 and |Fold Change| ≥ 1.5. Visual hierarchical cluster analysis by R software was used to show heatmaps and volcano plots (version 3.6.3). The squares of Pearson coefficient r values were calculated to show correlations between samples.

### Proteomic profiling and data processing

Liver tissues were grinded up in liquid nitrogen and then transferred to a 5-mL centrifuge tube. Then, 4-fold volumes of lysis buffer (8 mol/L urea, 1% Protease Inhibitor Cocktail) was added to the cell powder, followed by sonication three times on ice using a high intensity ultrasonic processor (Scientz). The remaining debris was removed by centrifugation at 12,000 × *g* at 4°C for 10 min. Finally, the supernatants were collected and the protein concentrations were determined with BCA kit according to the manufacturer’s instructions. The protein solution was digested to peptide with trypsin. Tryptic peptides were labeled by TMT kit/iTRAQ and fractionated into fractions by high pH reverse-phase HPLC. Then, tryptic peptides were dissolved in 0.1% formic acid (solvent A), directly loaded onto a home-made reversed-phase analytical column (15-cm length, 75 μm i.d.). The peptides were subjected to NSI source followed by tandem MS (MS/MS) in Q Exactive™ Plus (Thermo) coupled online to the ultra-performance liquid chromatography (UPLC). Peptides were then selected for MS/MS using NCE setting as 28 and the fragments were detected in the Orbitrap at a resolution of 17,500. The resulting MS/MS data were processed using Maxquant search engine (v.1.5.2.8). DEPs were calculated by the ratio of average expression between the two groups. Under the premise of *P* < 0.05 and |Fold Change| ≥ 1.3 was up-regulated and |Fold Change| < 1\1.3 was down-regulated. The squares of Pearson coefficient r values were calculated to show correlations between samples. Cluster membership were visualized by a heat map using the “heatmap.2” function from the “gplots” R-package.

### Metabolomic data processing

25 μL of plasma was added to a 96-well plate and then transferred to the Biomek 4000 workstation (Biomek 4000, Beckman Coulter, Inc., Brea, CA, USA). 100 μL ice-cold methanol with partial internal standards was automatically added to each sample and vortexed vigorously for 5 min. The plate was centrifuged at 4000 × *g* for 30 min. 30 μL of supernatants were transferred to a clean 96-well plate, and 20 μL of freshly prepared derivative reagents were added to each well. The plate was sealed and the derivatization was carried out at 30°C for 60 min. After derivatization, 350 μL of ice-cold 50% methanol solution was added to dilute the sample. Then the plate was stored at −20°C for 20 min and followed by 4000 × *g* centrifugation at 4°C for 30 min. 135 μL of supernatants were transferred to a new 96-well plate with 15 μL internal standards in each well. Serial dilutions of derivatized stock standards were added to the left wells. Finally, the plate was sealed for LC-MS analysis. A UPLC coupled to MS/MS (UPLC-MS/MS) system (ACQUITY UPLC-Xevo TQ-S, Waters Corp., Milford, MA, USA) was used to quantitate the microbial metabolite in this project. The raw data files generated by UPLC-MS/MS were processed using the QuanMET software (v2.0, Metabo-Profile, Shanghai, China) to perform peak integration, calibration and quantitation for each metabolite. The screening standard for changed metabolites was that the absolute value of |log_2_FC| > 0, *P* < 0.05 and FDR < 1. OPLS-DA, a multi-dimensional method, was used to further screen the differential metabolites. The screening criteria was Variable Importance in Projection (VIP) > 1. The changed metabolites obtained by T-test and OPLS-DA were intersected to identify metabolites with potential biological significance. Cluster membership were visualized by a heat map using the “heatmap.2” function from the “gplots” R-package. Combined with the relevant pathway associated metabolite sets (SMPDB) database, the biological functions of changed metabolites were analyzed.

### PCSF network

We built the network of metabolites and proteins with the support of the network model PCSF, using the interactions of protein–protein and protein–metabolite interactome as input prize for it. We then developed and re-arranged the whole network into several sub-networks with the information of clustergram. The molecular process was visualized with cytoscape (cytoscape.org).

### CNC network analysis

CNC networks were established utilizing co-expressed lncRNA-mRNA to obtain the interaction between lncRNAs and mRNAs. CNC networks were constructed with ten lncRNAs that were markedly changed in the livers of NASH mice and relevant DEGs. A Pearson correlation coefficient >0.95 was used as a cutoff to identify coding and non-coding genes.

### ceRNA network analysis

In order to identify potential miRNA response elements, three lncRNAs and thirteen miRNAs differentially expressed were subjected to ceRNA network analysis. Potential interactions were predicted by identifying and overlapping the same miRNA seed sequence binding site on both lncRNAs and mRNA sequences. TargetScan and miRanda were applied to predict potential targets of miRNAs.

### Functional enrichment analysis

GO annotation includes three categories: biological process, cellular compartment, and molecular function. GO analysis was used to demonstrate the specific biological function of gene or protein differentially expressed, and KEGG database was used to identify enriched pathways. GO annotation and KEGG pathways were analyzed by “clusterprofile” R-package. A corrected *P* value < 0.05 was considered significant enrichment.

### Patients

A total of 532 individuals, including healthy participants (*n* = 315), those with liver biopsy-proven NAFL (*n* = 110) and those with NASH (*n* = 107), were recruited at the First Affiliated Hospital of Wenzhou Medical University from December 2016 to February 2019. The baseline characteristics of these participants are shown in Supplementary Table S2. Written informed consent was obtained from each person. The diagnosis of NAFL and NASH was based on the histopathological features, including grade of steatosis, hepatocellular ballooning, and lobular inflammation. The NAS was calculated according to the Kleiner scoring system.

### MDA measurement

Hepatic MDA contents were measured by lipid peroxidation MDA assay kit (S0131, Beyotime Biotechnology, Shanghai, China) according to the manufacturer’s instructions.

### Hydroxyproline measurement

A hydroxyproline colorimetric assay kit (K555-100, BioVision, San Francisco, USA) was used to measure hepatic hydroxyproline levels according to the manufacturer’s instructions.

### Total RNA extraction and qRT-PCR

Total RNAs were extracted using TRIzol reagent (Invitrogen, CA, USA), and transcribed into first-strand complementary DNA (cDNA) using the Reverse Transcription System (Promega Corporation, Madison, WI, USA). The cDNAs were then used for qRT-PCR assays using SYBR Green Premix Ex Taq (Takara, Shiga, Japan) on Light Cycler 480 (Roche, Basel, Switzerland). Relative mRNA expression levels of target genes were normalized to the Rplp0 mRNA level and calculated using the cycle threshold (ΔΔCT). The primer sequences were listed in the Supplementary Table S3.

### Protein extraction and western blot

Liver tissues were lysed with radioimmunoprecipitation buffer containing 50 mmol/L Tris-HCl, 150mmol/L NaCl, 5mmol/L MgCl_2_, 2mmol/L EDTA, 1mmol/L NaF, 1% NP40, and 0.1% sodium dodecyl sulfate (SDS). The protein concentrations were quantified using a BCA protein assay kit (Thermo Fisher, CA, USA). Equal amounts of protein were subjected to SDS-PAGE electrophoresis and transferred onto nitrocellulose membranes. Membranes were blocked with 5% skimmed milk for 1 h at room temperature and then incubated with primary and secondary antibodies. The primary antibodies were used as follows: GDF3 antibody (1:1000, Catalog ab197390, Abcam, Cambridge, UK), PTGS2 antibody (1:2000, Catalog 12375-1-AP, Proteintech, Chicago, USA), SLC7A11 antibody (1:1000, Catalog 26864-1-AP, Proteintech), HMOX1 antibody (1:2000, Catalog 10701-1-AP, Proteintech), GPX4 antibody (1:5000, Catalog ab125066, Abcam), and α-Tubulin antibody (1:5000, Catalog 11224-1-AP, Proteintech).

### Data visualization and statistical analysis

All data were expressed as the means ± SEM except for the human characteristics in Supplementary Table S2, which is presented as mean ± SD or median (interquartile range). Two-tailed unpaired Student’s *t*-test was used to determine statistical differences between two groups. Significance between more than two groups was calculated using one-way ANOVA with Tukey’s post hoc test. For statistical correlation, Spearman correlation coefficient was used according to requirements. Venn diagram was performed with the BioVenn online tool (biovenn.nl/index.php). *P* values <0.05 were considered statistically significant. ^*^, ^**^, and ^***^ represent *P* < 0.05, *P* < 0.01, and *P* < 0.001, respectively. Other materials and bioinformatic software are described in Supplementary Material.

## Supplementary Material

loac034_suppl_Supplementary_Data_S1

loac034_suppl_Supplementary_Data_S2

loac034_suppl_Supplementary_Data_S3

loac034_suppl_Supplementary_Data_S4

loac034_suppl_Supplementary_Data_S5

loac034_suppl_Supplementary_Material

## Data Availability

All RNA-seq data have been deposited into the GEO database: GSE199121 and GSE189066. miRNA profile data have been deposited into the GEO database: GSE199246. LncRNA profile data have been deposited into the GEO database: GSE199306. The MS-based proteomic data have been deposited to the ProteomeXchange Consortium (proteomecentral.proteomexchange.org) via the iProX partner repository with the dataset identifier PXD032806.
