# Supplementary material for: A multi-omic landscape of steatosis-to-NASH progression
Source: Life Metab. 2022 Dec 1;1(3):242–57. doi: 10.1093/lifemeta/loac034 (PMC11749464; doi:10.1093/lifemeta/loac034)
Supplement: loac034_suppl_Supplementary_Material [file loac034_suppl_Supplementary_Material.docx]

**Supplementary Material**

**Supplementary Figure S1-S9, Supplementary Table S1-S3**

**
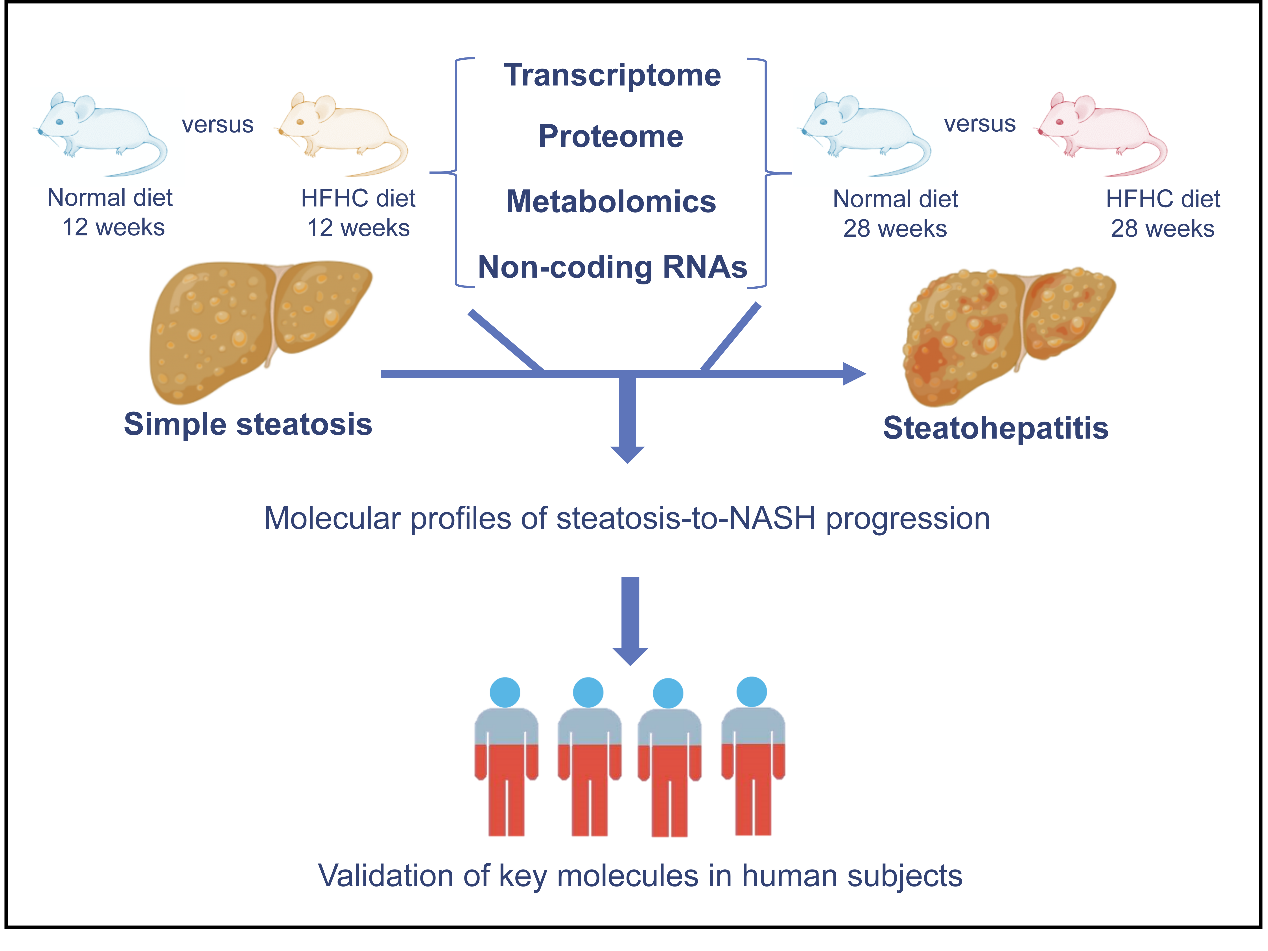
**

**Supplementary Figure S1. Outline of our current study.**

8-week-old C57BL/6 male mice were divided into two groups. In the first group, mice were fed a normal diet or HFHC diet for 12 weeks to induce simple steatosis. In the second groups, mice were fed a normal diet or HFHC diet for 28 weeks to induce NASH (n=6 in each group). The metabolic phenotypes and liver pathology have been described in our previous study [Liu B, Xiang L, Ji J, et al. J Clin Invest. 2021;131(20):e144801]. Multi-omic studies, including transcriptome, proteome, and plasma metabolomics, were performed and compared in mice with simple steatosis and NASH. Key findings were validated in human subjects and functional studies were performed *in vivo* and *in vitro*.

**
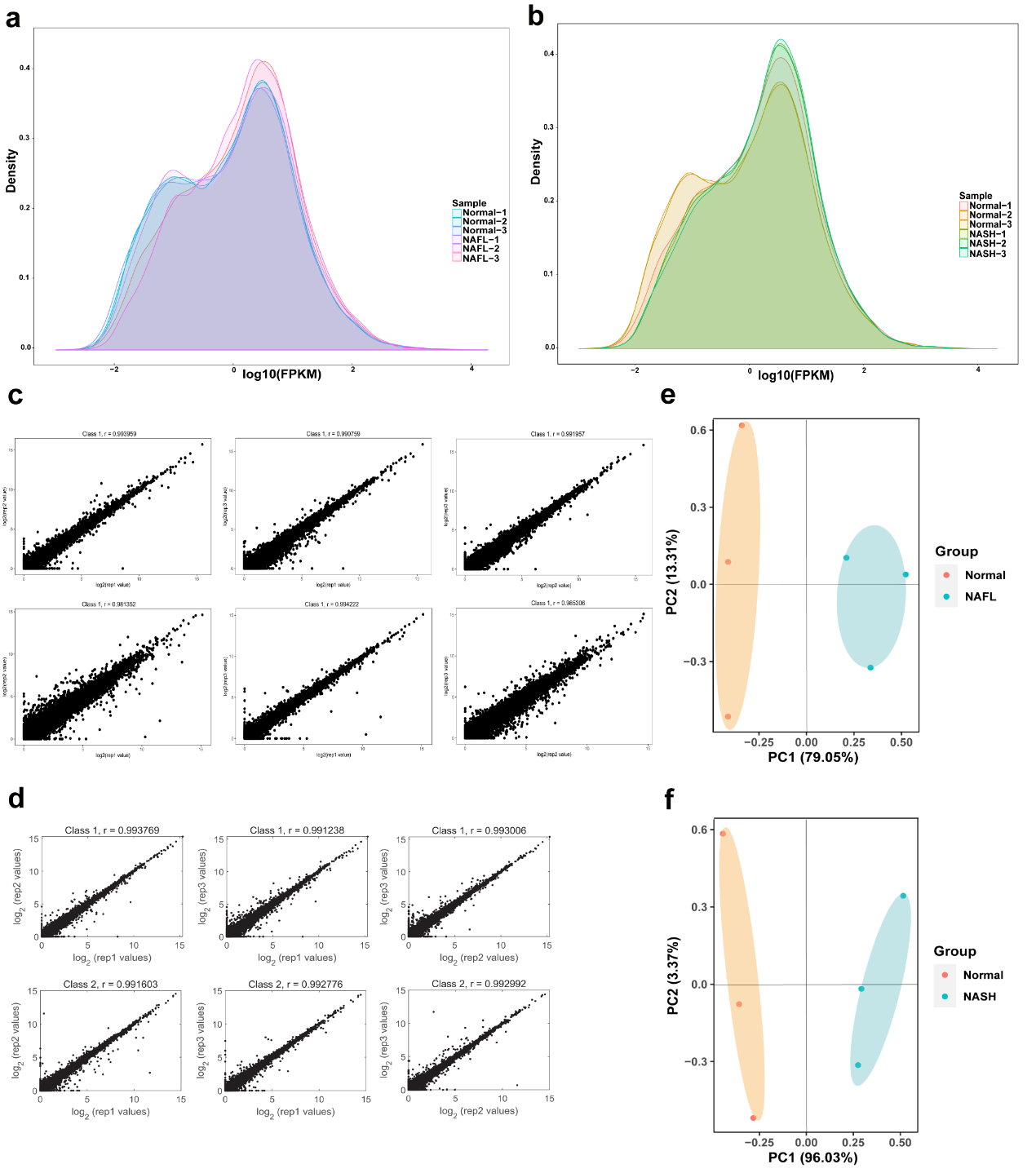
**

**Supplementary Figure S2. The reproductivity and reliability of transcriptomic datasets.**

**(a-b)** Gene expression density of transcriptomic profile in NAFL (a) and NASH mice (b) with their normal controls. **(c-d)** Spearman correlation analysis of transcriptomic profile in NAFL (c) and NASH mice (d) with their normal controls, respectively. **(e-f)** PCA analysis showing the normalized expression of genes in each mouse sample after batch-effect correction in NAFL (e) and NASH mice (f) with their age-matched normal controls, respectively.


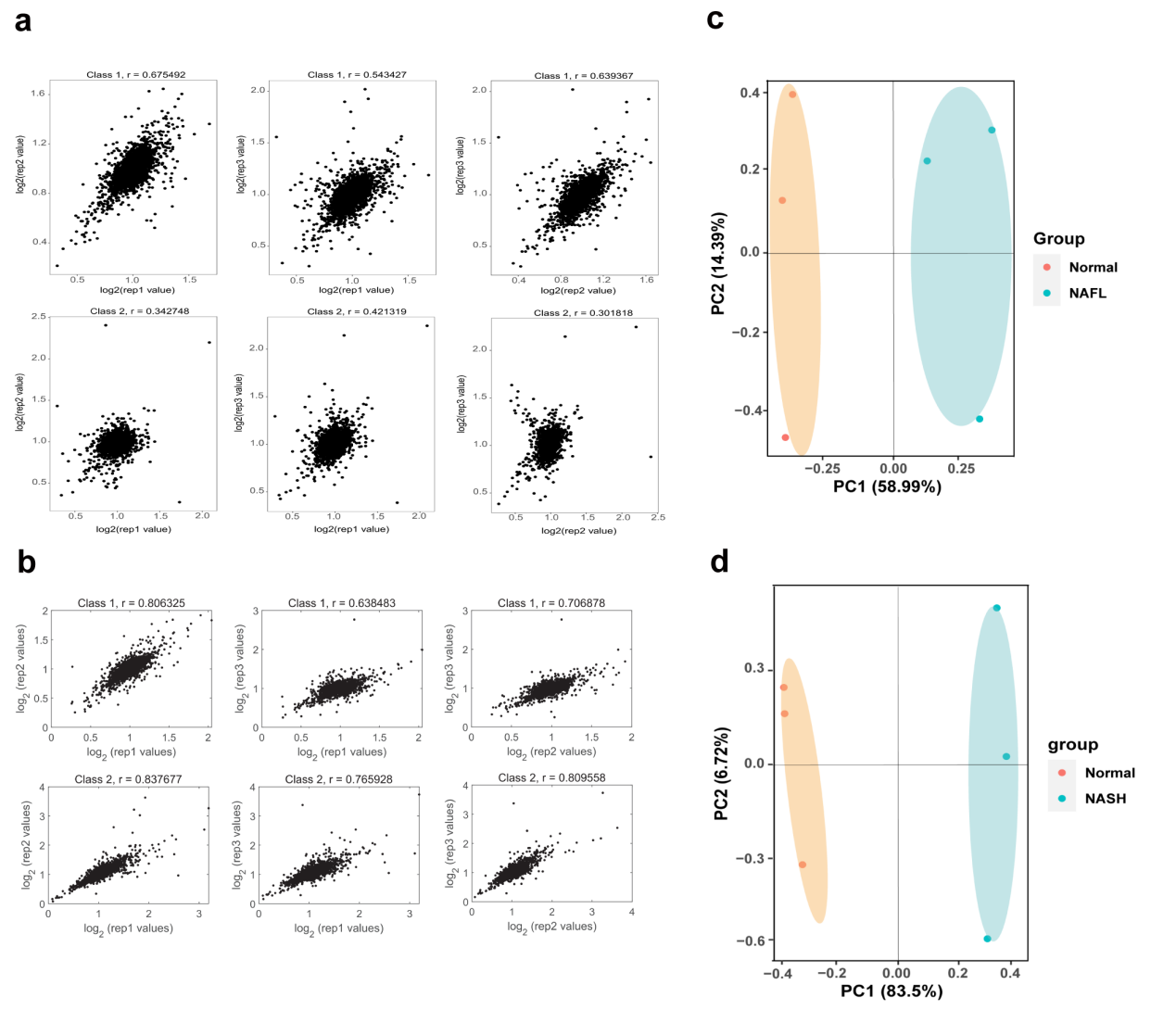


**Supplementary Figure S3. The reproductivity and reliability of proteomic datasets.**

**(a-b)** Spearman correlation analysis of proteomic profile in NAFL (a) and NASH (b) mice with their matched normal controls, respectively. **(c-d)** PCA Analysis showing the normalized expression of proteins in each mouse sample after batch-effect correction in NAFL (c) and NASH (d) mice with their age-matched normal controls, respectively.


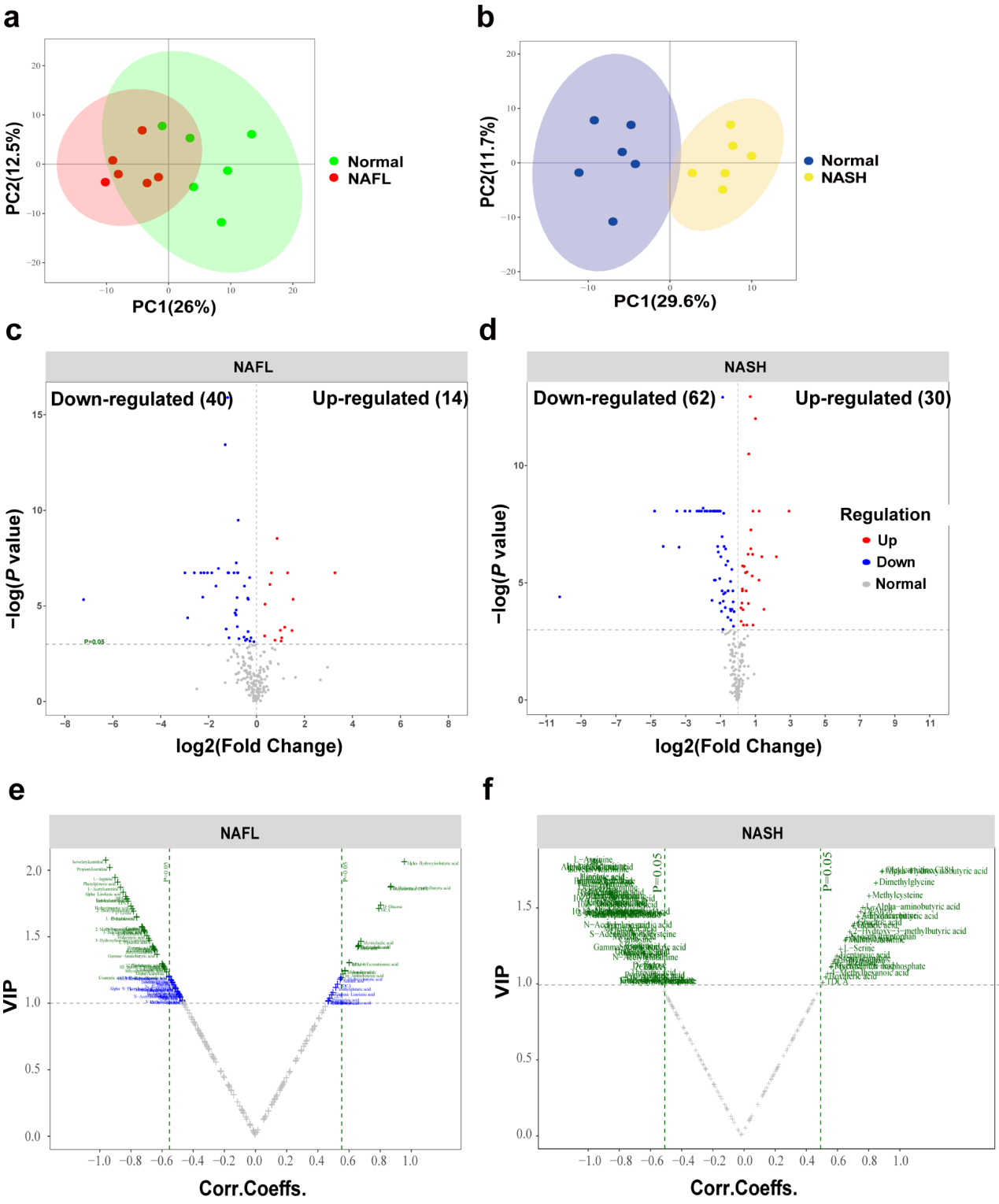


**Supplementary Figure S4. PCA and potential markers in plasma metabolomic profiles.**

**(a-b) PCA** analysis showing the normalized expression of proteins in each mouse sample after batch-effect correction in NAFL (a) and NASH (b) mice with their normal controls. **(c-d)** Volcano plot of dysregulated plasma metabolites calculated by Univariate analysis in NAFL (c) and NASH (d) mice with their normal controls. The bule circle dots represent down-regulated and the red circle dots represent up-regulated metabolites. **(e-f)** Volcano Plot of dysregulated plasma metabolites calculated by OPLS-DA analysis in NAFL (e) and NASH (f) mice with their normal controls. The green presents dysregulated metabolites [Variable Importance in Projection (VIP) > 1].


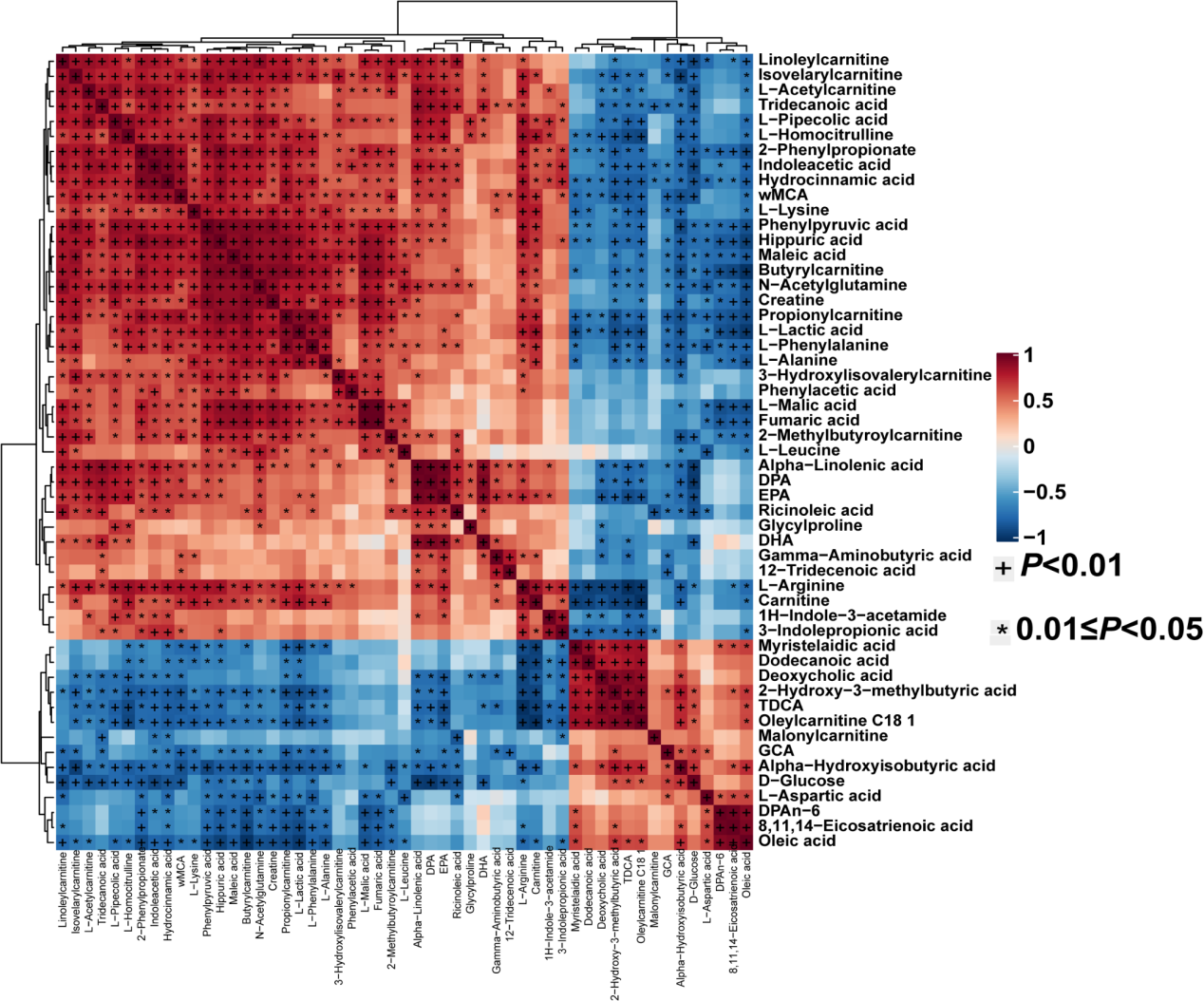


**Supplementary Figure S5. Correlation analysis of plasma metabolites in NAFL mice.**

Altered plasma metabolites between NAFL and normal mice were well-correlated.


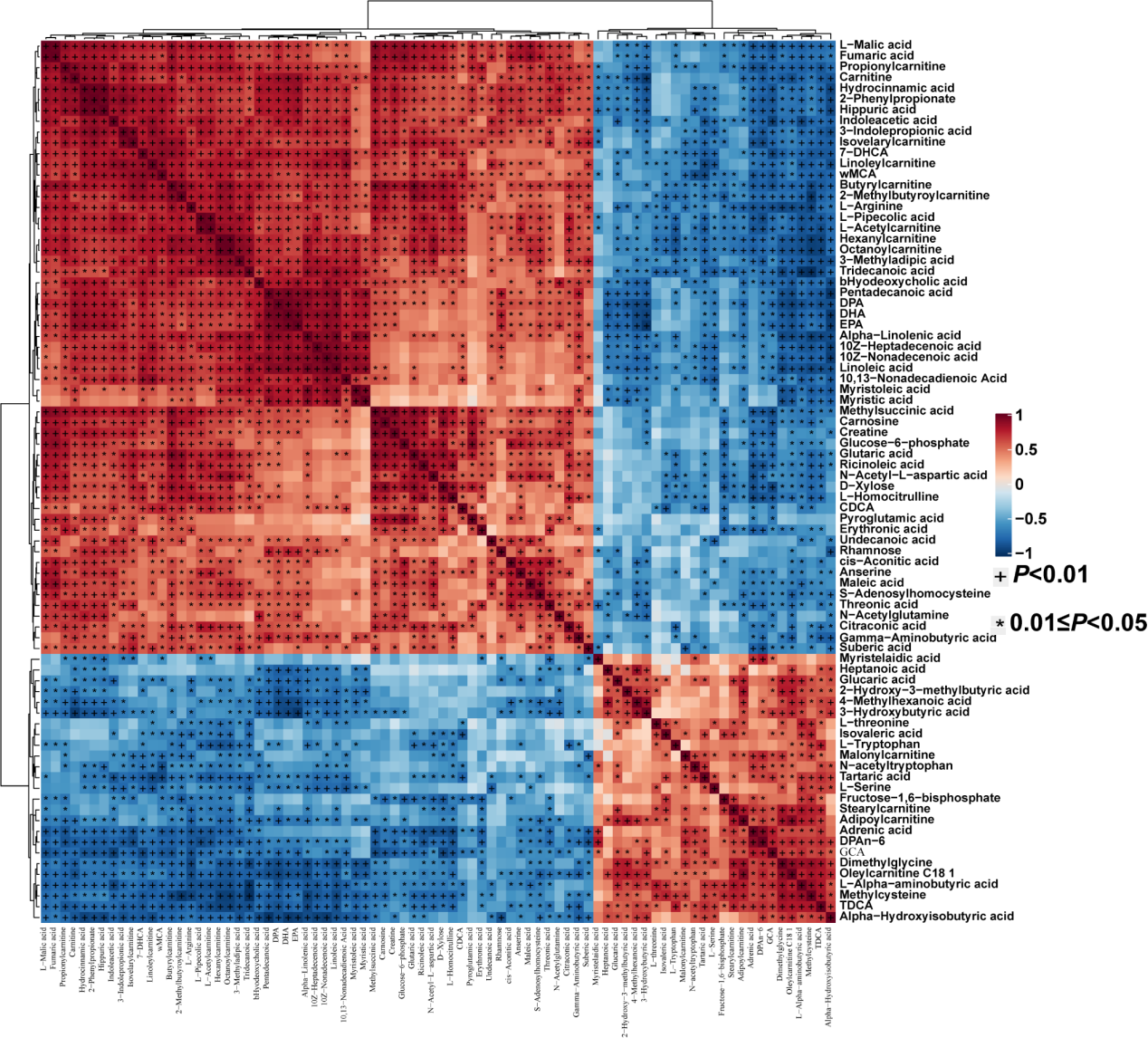


**Supplementary Figure S6. Correlation analysis of plasma metabolites in NASH mice.**

Altered plasma metabolites between NASH and normal mice were well-correlated.


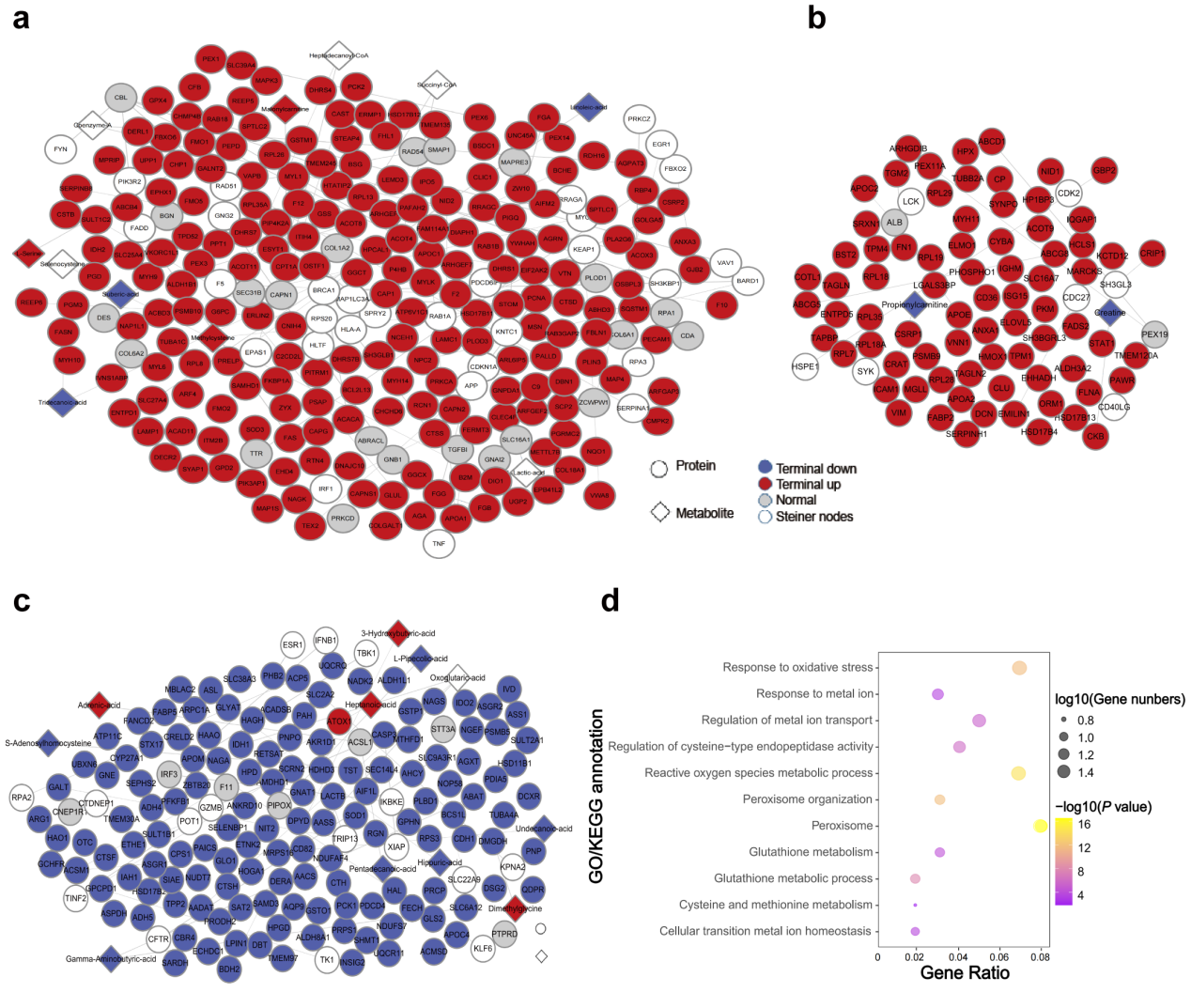


**Supplementary Figure S7. Integration of proteomic and metabolomics datasets in NASH.**

**(a)** Sub-network 2 in protein and metabolite interaction network. **(b)** Sub-network 3 in protein and metabolite interaction network. **(c)** Sub-network 5 in protein and metabolite interaction network. **(d)** GO and KEGG pathway analysis of proteins among protein and metabolite interaction network.

**
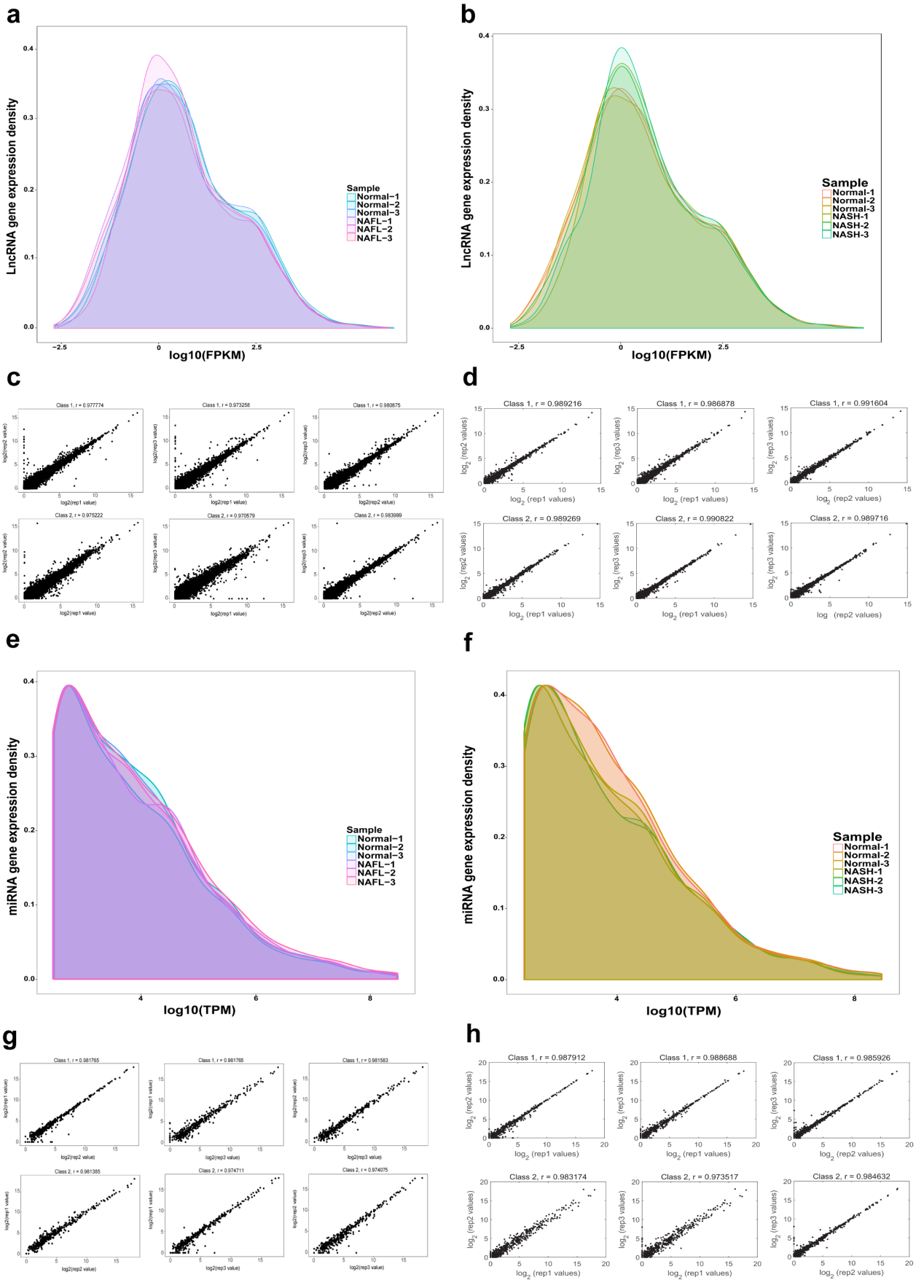
**

**Supplementary Figure S8. The reproductivity and reliability of lncRNA and miRNA datasets.**

**(a-b)** Gene expression density of lncRNA profile in NAFL (a) and NASH (b) mice with their matched normal controls. **(c-d)** Correlation analysis of lncRNA profile in NAFL (c) and NASH (d) mice with their normal controls. **(e-f)** Gene expression density of miRNA profile in NAFL (e) and NASH (f) mice with their normal controls. **(g-h)** Correlation analysis of miRNA profile in NAFL (g) and NASH (h) mice with their normal controls.


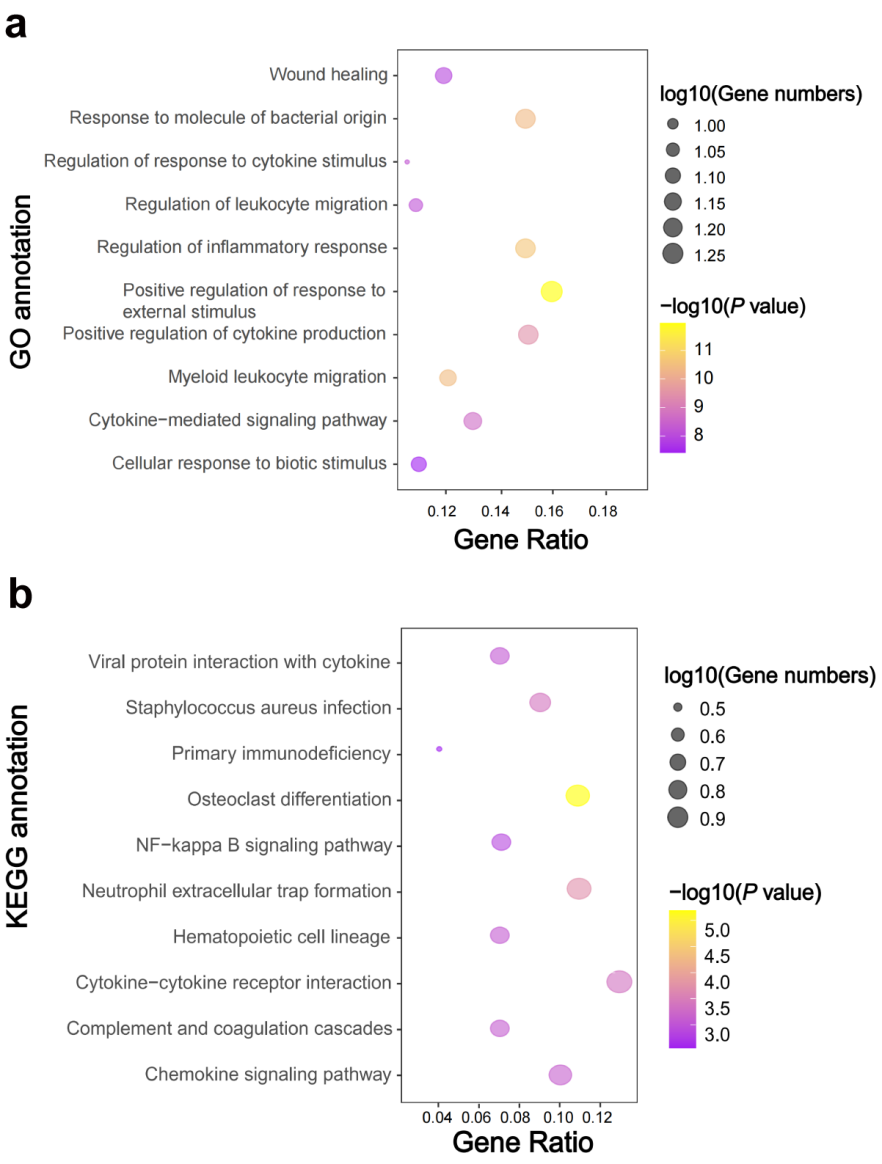


**Supplementary Figure S9. Biological process and KEGG pathway analysis of mRNAs predicted by CNC and ceRNA in NASH.**

**(a)** GO analysis of mRNAs predicted by CNC and ceRNA analysis. **(b)** KEGG pathway analysis of mRNAs predicted by CNC and ceRNA analysis.

**Supplementary Table
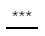

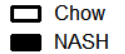
S1. mRNAs predicted by combining with CNC and ceRNA analysis in NASH.**

The genes identified in transcriptomic analysis were highlighted in bold.

| Gene name | *P* value | Log2FC | Regulation |
| --- | --- | --- | --- |
| ***Lck*** | 0.00137 | 1.26568 | up |
| ***Spi1*** | 1.29124E-49 | 2.15234 | up |
| ***Axl*** | 5.06651E-71 | 2.05495 | up |
| *Insig2* | 8.73914E-18 | 0.86376 | normal |
| ***Ctse*** | 0.00011 | 1.50612 | up |
| ***Cd33*** | 5.18432E-05 | 1.16665 | up |
| ***Corin*** | 3.92802E-06 | 2.10814 | up |
| ***Adamts4*** | 8.76767E-09 | 2.41051 | up |
| ***Tmem86a*** | 4.91202E-50 | 2.07114 | up |
| ***Lpl*** | 2.704E-206 | 3.15266 | up |
| ***Cd5l*** | 1.35855E-72 | 2.31987 | up |
| ***Fcgr1*** | 1.55938E-37 | 2.47155 | up |
| *Fli1* | 6.97262E-05 | 0.91856 | normal |
| ***Hsd11b1*** | 1.63013E-36 | -1.09737 | down |
| ***Slpi*** | 1.30392E-12 | 2.29615 | up |
| ***Pdk4*** | 0.00234 | 1.33209 | up |
| ***Btg2*** | 1.47436E-31 | 1.63228 | up |
| ***Cxcl13*** | 3.07045E-06 | -1.82477 | down |
| ***Clec4n*** | 4.21074E-17 | 1.82647 | up |
| ***Adam8*** | 5.04415E-37 | 4.58200 | up |
| *Adam23* | 0.00203 | 0.53304 | normal |
| ***Col3a1*** | 4.86964E-36 | 3.47950 | up |
| *Il1r1* | 0.05779 | 0.26857 | normal |
| *Stk17b* | 0.00043 | 0.50090 | normal |
| ***Tnfrsf11a*** | 1.45961E-10 | 1.86355 | up |
| ***Ptprc*** | 7.45463E-65 | 2.05871 | up |
| ***Soat1*** | 1.06196E-32 | 1.85216 | up |
| ***Rgs5*** | 2.13088E-39 | 1.43213 | up |
| *Crat* | 5.33193E-10 | 0.71293 | normal |
| *Usp20* | 0.00089 | 0.53124 | normal |
| *Lhx6* | 3.79222E-05 | 0.70454 | normal |
| ***Il1rn*** | 3.54247E-61 | 3.00230 | up |
| ***Prrg4*** | 1.35089E-19 | 2.14717 | up |
| *Skil* | 0.00057 | 0.56261 | normal |
| ***Anxa5*** | 7.7363E-127 | 2.24055 | up |
| ***Slc7a11*** | 4.10603E-13 | 3.40674 | up |
| ***Postn*** | 3.32737E-57 | 2.31431 | up |
| ***Tlr2*** | 3.67179E-28 | 2.20438 | up |
| ***Ifi44*** | 1.87732E-37 | 2.75701 | up |
| *Trp53inp1* | 0.01866 | 0.28207 | normal |
| ***Gbp3*** | 5.30457E-50 | 2.42133 | up |
| ***Aqp7*** | 1.11559E-13 | 3.45638 | up |
| ***Laptm5*** | 3.06877E-74 | 2.13589 | up |
| ***Ephb2*** | 1.88001E-93 | 6.81848 | up |
| ***Stap1*** | 1.79231E-14 | 1.92374 | up |
| ***Abcg3*** | 4.7701E-11 | 1.60341 | up |
| ***Spp1*** | 1.87047E-40 | 2.06645 | up |
| ***Cxcl9*** | 2.52778E-29 | 1.85816 | up |
| *Cyp3a25* | 4.03766E-06 | -0.42886 | normal |
| ***Eln*** | 1.22659E-15 | 2.27316 | up |
| *Tmem176b* | 5.06799E-17 | 0.83791 | normal |
| ***Arhgdib*** | 1.12733E-19 | 1.27617 | up |
| ***Cd9*** | 3.36335E-51 | 1.79777 | up |
| *Ctsc* | 3.40413E-17 | 0.84787 | normal |
| ***Tyrobp*** | 8.44023E-44 | 1.76504 | up |
| ***Il21r*** | 3.25093E-06 | 1.96600 | up |
| ***Itgax*** | 1.79239E-54 | 3.91206 | up |
| ***Fgf21*** | 5.32417E-20 | 2.08963 | up |
| *Acsm2* | -- | -- | -- |
| ***Plat*** | 4.38544E-17 | 3.32754 | up |
| ***Cx3cl1*** | 0.00040 | 1.30826 | up |
| *Katnb1* | 0.10775 | 0.42727 | normal |
| *Fam81a* | 0.02063 | -0.77026 | normal |
| ***Plscr4*** | 8.39068E-10 | 1.27983 | up |
| ***Nt5e*** | 1.01546E-08 | 1.16591 | up |
| *Gng11* | 0.00191 | 0.56772 | normal |
| ***Casp4*** | 7.91989E-05 | 1.26682 | up |
| *Gabrb3* | 0.00383 | 0.77531 | normal |
| ***Plcg2*** | 5.80982E-14 | 1.65056 | up |
| *Hsd17b13* | 1.05536E-16 | 0.70010 | normal |
| *Sema3f* | 6.2118E-07 | 0.77127 | normal |
| *Eogt* | 0.00018 | 0.65473 | normal |
| ***Isg15*** | 3.01827E-21 | 1.84267 | up |
| *P2ry12* | 0.00852 | 0.76756 | normal |
| ***Spon2*** | 2.73365E-33 | 2.30058 | up |
| ***Serpine1*** | 1.1343E-26 | 3.18225 | up |
| ***Tnfrsf23*** | 3.08755E-13 | 2.51410 | up |
| ***Dock10*** | 1.31521E-30 | 1.84940 | up |
| ***Dusp10*** | 3.2287E-06 | 1.13999 | up |
| ***Mmd2*** | 1.47734E-13 | 2.10200 | up |
| *Pnrc1* | 0.98495 | 0.00296 | normal |
| ***Gbp2b*** | 5.62194E-06 | 1.30553 | up |
| ***C3ar1*** | 2.15396E-18 | 1.88748 | up |
| ***Cd53*** | 9.21814E-44 | 1.91830 | up |
| ***Slc35f2*** | 1.3326E-20 | 2.50229 | up |
| ***Mab21l3*** | 3.4912E-12 | 3.24116 | up |
| ***Defb1*** | 1.61891E-07 | 1.77715 | up |
| ***Napepld*** | 4.94368E-06 | 1.36724 | up |
| ***Cxcr4*** | 3.26816E-16 | 2.13699 | up |
| ***Hcar2*** | 3.05983E-11 | 2.60912 | up |
| *Slc25a51* | 0.01402 | -0.34023 | normal |
| ***Kcnk6*** | 1.74302E-14 | 1.51141 | up |
| *Atp10d* | 0.00926 | 0.54203 | normal |
| ***Selplg*** | 4.12821E-24 | 2.10422 | up |
| ***Lhfp*** | 1.10375E-17 | 1.12127 | up |
| ***C5ar1*** | 8.9184E-24 | 2.34665 | up |
| ***Vsig8*** | 2.40686E-19 | 4.02115 | up |
| ***Lrrc25*** | 5.83212E-18 | 1.67673 | up |
| ***Mmp13*** | 1.7583E-38 | 5.05261 | up |
| ***Clec12a*** | 1.10258E-43 | 2.28920 | up |
| *Cd8a* | -- | -- | -- |
| *Mmrn1* | -- | -- | -- |
| *Tead1* | 0.009616794 | 0.51327 | normal |
| *Tmem154* | -- | -- | -- |
| *Gjc3* | 0.246692439 | -0.37924 | normal |
| ***Sema3b*** | 2.9164E-22 | 2.86842 | up |
| ***Cxcl2*** | 1.19635E-09 | 2.72584 | up |
| ***Fcgr4*** | 3.07069E-31 | 1.81614 | up |
| ***Fabp4*** | 1.37625E-83 | 2.10899 | up |
| *Amn1* | 0.236909005 | 0.21443 | normal |
| ***Saa1*** | 1.5936E-259 | 3.41017 | up |
| *Nlrc5* | 0.000463317 | 0.46661 | normal |
| ***Clec7a*** | 3.0614E-111 | 3.49803 | up |
| ***Btc*** | 7.56298E-16 | 2.10209 | up |
| *Rasgef1b* | 8.98157E-07 | 0.66188 | normal |
| ***Cyp2a22*** | 1.50303E-44 | 2.79726 | up |

**Supplementary Table S2. Clinical characteristics of patients with NAFLD and normal individuals**

| Variables | Normal | NAFL | NASH | P-value | P-value§ |
| --- | --- | --- | --- | --- | --- |
| Sample size | 315 | 110 | 107 | - | - |
| Age (years) | 43.05±11.94 | 42.32±11.20 | 42.47±10.95 | - | - |
| Gender (male n, %) | 252 (80.3) | 89 (80.9) | 86 (80.4) | - | - |
| BMI (kg/m^2^) | 22.0(20.3-24.0) | 26.0(24.5-28.0) | 27.1(24.6-29.4) | - | - |
| Systolic BP (mmHg) | 112.8±11.6 | 127.9±15.1 | 129.8±14.0 | <0.001 | <0.0001 |
| Diastolic BP (mmHg) | 75.6±8.5 | 83.5±10.0 | 84.6±10.4 | <0.0001 | <0.0001 |
| Triglycerides (mmol/L) ^a^ | 0.90(0.72-1.19) | 1.78(1.18-2.48) | 2.18(1.63-3.10) | <0.0001 | <0.0001 |
| Total cholesterol (mmol/L) | 4.48±0.62 | 4.85±4.14 | 5.61±1.26 | <0.0001 | <0.0001 |
| LDL-c (mmol/L) | 2.51±0.53 | 2.83±0.78 | 3.24±1.02 | <0.0001 | <0.001 |
| HDL-c (mmol/L) | 1.55±0.38 | 0.99±0.22 | 0.99±0.20 | <0.0001 | <0.0001 |
| FPG (mmol/L) ^a^ | 4.7(4.4-5.0) | 5.2(4.9-6.0) | 5.4(4.9-6.2) | <0.0001 | <0.0001 |
| HbA1c (%) ^a^ | 5.3(5.1-5.4) | 5.8(5.4-6.6) | 5.9(5.4-6.6) | <0.0001 | <0.0001 |
| HOMA-IR | 0.79(0.60-1.04) | 1.50(1.00-2.31) | 2.20(1.38-3.06) | <0.0001 | <0.0001 |
| ALT (U/L) ^a^ | 15(12-20) | 38(25-60) | 61(38-90) | <0.0001 | <0.0001 |
| AST (U/L) ^a^ | 19(17-23) | 28(23-35) | 38(27-56) | <0.0001 | <0.0001 |
| ALP (U/L) ^a^ | 60(49-72) | 74(65-92) | 85(70-96) | <0.0001 | <0.0001 |
| GGT (U/L) ^a^ | 15(10-21) | 44(27-74) | 58(41-109) | <0.0001 | <0.0001 |
| Hepatocellular Ballooning | - | 0.64±0.60 | 1.54±0.50 | <0.0001 | <0.0001 |
| Steatosis | - | 1.51±0.66 | 2.14±0.72 | <0.0001 | <0.0001 |
| Lobular Inﬂammation | - | 0.75±0.53 | 1.44±0.54 | <0.0001 | <0.0001 |

BMI=body mass index; BP=blood pressure; LDL-c=low density lipoprotein cholesterol; HDL-c=high density lipoprotein cholesterol; FPG=fasting plasma glucose; HbA1c= glycated hemoglobin; HOMA-IR=homeostasis model assessment of insulin resistance; ALT=alanine transaminase; AST=aspartate aminotransferase; ALP=alkaline phosphatase; GGT=γ-glutamyl transpeptidase; Data are presented as the mean ± SD or median (interquartile range).

^a^: Analysis performed on log-transformed data. §: adjusted for age, gender and BMI.

**Supplementary
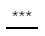

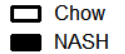
Table S3. Primer sequences for quantitative real-time PCR analysis**

| Gene | | Sequence 5’→3’ | |
| --- | --- | --- | --- |
| Mouse *Srebp-1c* | | F：CAAGGCCATCGACTACATCCG | |
|  |  | R：CACCACTTCGGGTTTCATGC | |
| Mouse *Fasn* | | F：GGAGGTGGTGATAGCCGGTAT | |
|  |  | R: TGGGTAATCCATAGAGCCCAG | |
| Mouse *Scd1* | | F：TTCTTGCGATACACTCTGGTGC | |
|  |  | R：CGGGATTGAATGTTCTTGTCGT | |
| Mouse *Acc1* | | F：GATGAACCATCTCCGTTGGC | |
|  |  | R：GACCCAATTATGAATCGGGAGTG | |
| Mouse *Pparα* | | F：TTTCGGCGAACTATTCGGCTG | |
|  |  | R：GGCATTTGTTCCGGTTCTTCTT | |
| Mouse *Cpt1α* | | F：AGATCAATCGGACCCTAGACAC | |
|  |  | R：CAGCGAGTAGCGCATAGTCA | |
| Mouse *Mcad* | | F：CCAGAGAGGAGATTATCCCCG | |
|  |  | R：TACACCCATACGCCAACTCTT | |
| Mouse *Acox1* | | F：CCGCCACCTTCAATCCAGAG | |
|  |  | R：CAAGTTCTCGATTTCTCGACGG | |
| Mouse *Cd36* | | F：ATGGGCTGTGATCGGAACTG | |
|  |  | R：ATGGGCTGTGATCGGAACTG | |
| Mouse *Tnf* | | F：AGCCCCCAGTCTGTATCCTT | |
|  |  | R：CTCCCTTTGCAGAACTCAGG | |
| Mouse *IL 1b* | | F：TGGCAACTGTTCCTGAACTCAA | |
|  |  | R：AGCAGCCCTTCATCTTTTGG | |
| Mouse *IL6* | | F：AGTTGCCTTCTTGGGACTGA | |
|  |  | R：TCCACGATTTCCCAGAGAAC | |
| Mouse *IL12b* | | F：CCAGAGACATGGAGTCATAG | |
|  |  | R：AGATGTGAGTGGCTCAGAGT | |
| Mouse *Nos2* | | F：GAGGCCCAGGAGGAGAGAGATCCG | |
|  |  | R：TCCATGCAGACAACCTTGGTGTTG | |
| Mouse *Ccl2* | | F：AGGTCCCTGTCATGCTTCTG | |
|  |  | R：TCTGGACCCATTCCTTCTTG | |
| Mouse *Ccl5* | | F：TGCCCACGTCAAGGAGTATTT | |
|  |  | R：TTCTCTGGGTTGGCACACACT | |
| Mouse *Adgre1* | | F：ATCCTTGGCCATCCGGCAGA | |
|  |  | R：GCAAAGCCAGGGTGGCAAGT | |
| Mouse *Col1a1* | | F：AAGAGGCGAGAGAGGTTTCC | |
|  |  | R：AGAACCATCAGCACCTTTGG | |
| Mouse *Col3a1* | | F：CTGTAACATGGAAACTGGGGAAA | |
|  |  | R：CCATAGCTGAACTGAAAACCACC | |
| Mouse *Acta2* | | F：CTGACAGAGGCACCACTGAA | |
|  |  | R：CATCTCCAGAGTCCAGCACA | |
| Mouse *Tgfb1* | | F：ACCATGCCAACTTCTGTCTGGGAC | |
|  |  | R：ACAACTGCTCCACCTTGGGCTTG | |
| Mouse *Mmp13* | | F：TGCTTCCTGATGATGACGTTCAAGG | |
|  |  | R：TGGGATGCTTAGGGTTGGGGTC | |
| Mouse *36B4* | | F：GAAACTGCTGCCTCACATCCG | |
|  |  | R：GCTGGCACAGTGACCTCACACG | |
| Mouse *Gdf3* | | F：ATGCAGCCTTATCAACGGCTT | |
|  |  | R：AGGCGCTTTCTCTAATCCCAG | |
| Mouse *Trem2* | | F：CTGGAACCGTCACCATCACTC | |
|  |  | R：CGAAACTCGATGACTCCTCGG | |
| Mouse *Lcn2* | | F：TGGCCCTGAGTGTCATGTG | |
|  |  | R：CTCTTGTAGCTCATAGATGGTGC | |
| Mouse *Ephb2* | | F：GCGGCTACGACGAGAACAT | |
|  |  | R：GGCTAAGTCAAAATCAGCCTCA | |
| Mouse *Mmp12* | | F：CTGCTCCCATGAATGACAGTG | |
|  |  | R：AGTTGCTTCTAGCCCAAAGAAC | |
| Mouse *Sprr1a* | | F：TTGTGCCCCCAAAACCAAG | |
|  |  | R：GGCTCTGGTGCCTTAGGTTG | |
| Mouse *Hsbp1* | | F：GAGACGGACCCCAAGACCA | |
|  |  | R：GGTCGTCAATCCGACTGCT | |
| Mouse *Hmox1* | | F：AAGCCGAGAATGCTGAGTTCA | |
|  |  | R：GCCGTGTAGATATGGTACAAGGA | |
| Mouse *Cisd1* | | F：GCTGTGCGAGTTGAGTGGAT | |
|  |  | R：TGGTGCGATTCTCTTTAGCGTA | |
| Mouse *Fancd2* | | F：CAAAATCAGCTAGGTGTGGATCA | |
|  |  | R：CCAGGCCATTAACAAACTCTTCT | |
| Mouse *Lpcat3* | | F：GACGGGGACATGGGAGAGA | |
|  |  | R：GTAAAACAGAGCCAACGGGTAG | |
| Mouse *Pebp1* | | F：CCAGCAGCATTTCATGGGAC | |
|  |  | R：TGGTGCCACTCCCTGAATTTG | |
| Mouse *Zeb1* | | F：GCTGGCAAGACAACGTGAAAG | |
|  |  | R：GCCTCAGGATAAATGACGGC | |
| Mouse *Fdft1* | | F：ATGGAGTTCGTCAAGTGTCTAGG | |
|  |  | R：CGTGCCGTATGTCCCCATC | |
| Mouse *Sqle* | | F：ATAAGAAATGCGGGGATGTCAC | |
|  |  | R：ATATCCGAGAAGGCAGCGAAC | |
| Mouse *Fads2* | | F：AAGGGAGGTAACCAGGGAGAG | |
|  |  | R：CCGCTGGGACCATTTGGTAA | |
| Mouse *Nfe2l2* | | F：CCCAGCAGGACATGGATTTGA | |
|  |  | R：AGCTCATAGTCCTTCTGTCGC | |
| Mouse *Nqo1* | | F：AGGATGGGAGGTACTCGAATC | |
|  |  | R：AGGCGTCCTTCCTTATATGCTA | |
| Mouse *Slc7a11* | | F：GGCACCGTCATCGGATCAG | |
|  |  | R：CTCCACAGGCAGACCAGAAAA | |
| Mouse *Gclc* | | F：GGGGTGACGAGGTGGAGTA | |
|  |  | R：GTTGGGGTTTGTCCTCTCCC | |
| Mouse *Abcc1* | | F：TGCAGAGGCATCTCAGCAAC | |
|  |  | R：TCCTCCTTAGCTCCAGCCTT | |
| Mouse *Slc1a5* | | F：TCACCATCCTGGTCACAGC | |
|  |  | R：ACCTTCCACGTTGAGGACAG | |
| Mouse *Pgd* | | F：TGAAGGGTCCTAAGGTGGTCC | |
|  |  | R：CCGCCATAATTGAGGGTCCAG | |
| Mouse *Gpx4* | | F：GATGGAGCCCATTCCTGAACC | |
|  |  | R：CCCTGTACTTATCCAGGCAGA | |
| Mouse *Fth1* | | F：CAAGTGCGCCAGAACTACCA | |
|  |  | R：GCCACATCATCTCGGTCAAAA | |
| Mouse *Ptgs2* | | F：TGAGCAACTATTCCAAACCAGC | |
|  |  | R：GCACGTAGTCTTCGATCACTATC | |

**Supplementary Materials**

**Key Resources Table**

| REAGENT or RESOURCE | SOURCE | IDENTIFIER |
| --- | --- | --- |
| Antibodies |  |  |
| GDF3 antibody | Abcam (Cambridge, UK) | Cat#ab197390 |
| PTGS2 antibody | Proteintech (Chicago, USA) | Cat#12375-1-AP |
| SLC7A11 antibody | Proteintech (Chicago, USA) | Cat#26864-1-AP |
| HMOX1 antibody | Proteintech (Chicago, USA) | Cat#10701-1-AP |
| GPX4 antibody | Abcam (Cambridge, UK) | Cat#ab125066 |
| α-Tubulin antibody | Proteintech (Chicago, USA) | Cat# 11224-1-AP |
| Biological samples |  |  |
| Human plasma samples  and clinical information | First Affiliated Hospital of Wenzhou Medical University (Wenzhou, China) | Supplementary Table 2 |
| Chemicals and Recombinant Proteins |  |  |
| Zinc protoporphyrin IX (ZnPP) | MedChemExpress (New Jersey, USA) | Cat#15442-64-5, |
| Recombination GDF3 protein | R&D systems (Minnesota, USA) | Cat#9009-GD-010 |
| TRIzol reagent | Thermo Fisher Scientific (California, USA) | Cat#10296010 |
| PrimeScript RT Master Mix | Takara (Shiga, Japan) | Cat#RR036A |
| SYBR Premix Ex TaqDRR041A | Takara (Shiga, Japan) | Cat#DRR820A |
| 4 × Protein SDS PAGE Loading Buffer | Takara (Shiga, Japan) | Cat#9173 |
| RIPA lysis buffer | Beyotime Biotechnology (Shanghai, China) | Cat#P0013B |
| SDS-PAGE Gel Quick Preparation Kit | Beyotime Biotechnology (Shanghai, China) | Cat#P0012AC |
| Nonidet P40 | Thermo Fisher Scientific (California, USA) | Cat#85124 |
| NEBNext Ultra II RNA Library Prep Kit for Illumina | NEB (USA) | Cat#E7770 |
| Protease Inhibitor Cocktails | MedChemExpress (New Jersey, USA) | Cat#HY-K0011 |
| Bovine Serum Albumin | Millipo (Massachusetts, USA) | Cat#126575 |
| Dulbecco's Modified Eagle Medium | gbico | Cat#12995-065 |
| DMEM-F12 | gbico | Cat#C11330500BT |
| DPBS | Servicebio (Shanghai, China) | Cat#G4200 |
| Immobilon-P® Transfer Membrane (PVDF) | Millipo (Massachusetts, USA) | Cat#IPVH00010 |
| Methanol | Thermo Fisher Scientific (California, USA) | Cat#A454K4 |
| Critical Commercial Assays |  |  |
| Lipid peroxidation MDA assay kits | Beyotime Biotechnology (Shanghai, China) | Cat#S0131 |
| Hydroxyproline colorimetric assay kit | BioVision (San Francisco, USA) | Cat#K555- 100 |
| BCA protein assay kit | Thermo Fisher Scientific (California, USA) | Cat#23225 |
| Triglyceride assay kit | Ke Hua Biotechnology (Shanghai, China) | N/A |
| Mouse GDF3 assay kit | Raybiotech (Atlanta, USA) | Cat#ELM-GDF3 |
| Human GDF3 assay kit | Raybiotech (Atlanta, USA) | Cat#ELH-GDF3 |
| Diet |  |  |
| Normal diet (ND) | Shanghai Laboratory Animal company (SLAC, Shanghai, China) | N/A |
| High-fat high-cholesterol high-fructose diet (HFHC diet) | Research Diets Inc (New Brunswick, USA) | Cat#D09100310 |
| Choline-deficient high fat diet (CD-HFD) | Research Diets Inc (New Brunswick, USA) | Cat#A06071302 |
| Methionine and choline-deficient diet (MCD) | Research Diets Inc (New Brunswick, USA) | Cat#A02082002BR |
| Deposited data |  |  |
| Raw and preprocessed transcriptomics data | this paper | Gene Expression Omnibus: GSE199121 and GSE189066. |
| Raw and preprocessed proteomics data | this paper | iProX database with accession number: PXD032806 |
| Raw and preprocessed lncRNA profile data | this paper | Gene Expression Omnibus: GSE199306 |
| Raw and preprocessed miRNA profile data | this paper | Gene Expression Omnibus: GSE199246 |
| Experimental Models: Cell Lines |  |  |
| AML12 cell line | Cell Bank of Shanghai Institutes of Biological Sciences | Serial#SCSP-550 |
| RAW 264.7 cell line | Cell Bank of Shanghai Institutes of Biological Sciences | Serial#TCM13 |
| Experimental Models: Organisms |  |  |
| Mouse: C57BL/6J | Shanghai Laboratory Animal Company (SLAC, Shanghai) | N/A |
| Oligonucleotides |  |  |
| Primers used for real-time quantitative PCR | Supplementary Table 3 | N/A |
| Software and algorithms |  |  |
| GraphPad Prism v 9.0 | GraphPad Software Inc | https://www.graphpad.com/scientifific-software/prism |
| R v 3.4.1 | R Core Team | https://www.r-project.org/ |
| R Studio | RStudio Team | https://www.rstudio.com/ |
| FastQC | Babraham Bioinformatics Institute | https://www.bioinformatics.babraham.ac.uk/projects/fastqc/ |
| DESeq2 | Anders S, Huber W [1]. | https://www.bioconductor.org/packages/release/bioc/html/DESeq2.html |
| pheatmap | R Core Team | https://cran.r-project.org/web/ packages/pheatmap/index.html |
| ggplot2 | R Core Team | https://cran.r-project.org/web/packages/ggplot2/index.html |
| TopHat | Kim D, Pertea G, Trapnell C, Pimentel H, Kelley R, Salzberg SL [2]. | N/A |
| Bowtie | Langmead B. and S. L. Salzberg [3]. | N/A |
| Cufflinks | Trapnell C, Williams BA, Pertea G, Mortazavi A, et al [4]. | N/A |
| QuanMET | Metabo-Profile (Shanghai, China) | N/A |
| KEGG | Kanehisa M, Goto S, Kawashima S, Okuno Y, et al [5]. | https://www.kegg.jp/ |
| goseq | Young MD, Wakefield MJ, Smyth GK, Oshlack A, et al [6]. | https://www.bioconductor.org/packages/release/bioc/html/goseq.html |
| Cytoscape | Shannon P, Markiel A, Ozier O, et al [7]. | https://cytoscape.org/ |
| MaxQuant | Cox J, Mann M [8]. | http://www.maxquant.org/ |
| TargetScan | Riffo-Campos ÁL, Riquelme I, Brebi-Mieville P [9]. | http://www.targetscan.org/ |
| miRanda | Riffo-Campos ÁL, Riquelme I, Brebi-Mieville P [9]. | http://www.mircorna.org/ |
| Prize-collecting Steiner Forest (PCSF) network model | Pirhaji L, Milani P, Leidl M, et al [10]. | N/A |
| Data Availability |  |  |
| Raw and preprocessed transcriptomics data | this paper | Gene Expression Omnibus: GSE199121 and GSE189066.  (https://www.ncbi.nlm.nih.gov/geo/query/acc.cgi?acc=GSE199121; https://www.ncbi.nlm.nih.gov/geo/query/acc.cgi?acc=GSE189066) |
| Raw and preprocessed proteomics data | this paper | iProX database with accession number: PXD032806 |
| Raw and preprocessed lncRNA profile data | this paper | Gene Expression Omnibus: GSE199306  (https://www.ncbi.nlm.nih.gov/geo/query/acc.cgi?acc=GSE199306) |
| Raw and preprocessed miRNA profile data | this paper | Gene Expression Omnibus: GSE199246  (https://www.ncbi.nlm.nih.gov/geo/query/acc.cgi?acc=GSE199246) |
| NASH patients’ transcriptomics data | Gene Expression Omnibus DataSets | Gene Expression Omnibus: GSE89632 (https://www.ncbi.nlm.nih.gov/geo/query/acc.cgi?acc=GSE89632) |
| NASH patients’ transcriptomics data | Gene Expression Omnibus DataSets | Gene Expression Omnibus: GSE24807 (https://www.ncbi.nlm.nih.gov/geo/query/acc.cgi?acc=GSE24807) |
| Mouse secretome database | Gene Expression Omnibus DataSets | Gene Expression Omnibus: GSE10246 (https://www.ncbi.nlm.nih.gov/geo/query/acc.cgi?acc=GSE10246) |
| Single-cell transcriptomics data | Gene Expression Omnibus DataSets | Gene Expression Omnibus: GSE129516 (https://www.ncbi.nlm.nih.gov/geo/query/acc.cgi?acc=GSE129516) |

**References**

1. Anders S, Huber W. Differential expression analysis for sequence count data. Genome Biol 2010;**11**:R106.
2. Kim D, Pertea G, Trapnell C et al. TopHat2: accurate alignment of transcriptomes in the presence of insertions, deletions and gene fusions. Genome Biol 2013;**14**:R36.
3. Langmead B, Salzberg SL. Fast gapped-read alignment with Bowtie 2. Nat. Methods 2012;**9**:357-359.
4. Trapnell C, Williams BA, Pertea G et al. Transcript assembly and quantification by RNA-Seq reveals unannotated transcripts and isoform switching during cell differentiation. Nat. Biotechnol 2010;**28**:511-515.
5. Kanehisa M, Goto S, Kawashima S et al. The KEGG resource for deciphering the genome. Nucleic Acids Res 2004;**32**:277-280.
6. Young MD, Wakefield MJ, Smyth GK et al. Gene ontology analysis for RNA-seq: accounting for selection bias. Genome Biol 2010;**11**:R14.
7. Shannon P, Markiel A, Ozier O et al. Cytoscape: a software environment for integrated models of biomolecular interaction networks. Genome Res 2003;**13**:2498-2504.
8. Cox J, Mann M. MaxQuant enables high peptide identification rates, individualized p.p.b.-range mass accuracies and proteome-wide protein quantification. Nat. Biotechnol 2008;**26**:1367-1372.

9. Riffo-Campos ÁL, Riquelme I, Brebi-Mieville P. Tools for sequence-based miRNA target prediction: what to choose? Int. J. Mol. Sci 2016;**17**:1987.

10. Pirhaji L, Milani P, Leidl M et al. Revealing disease-associated pathways by network integration of untargeted metabolomics. Nat.Methods 2016;**13**:770-776.
